# Photochemical Action Plots Map Orthogonal Reactivity in Photochemical Release Systems

**DOI:** 10.1002/advs.202402011

**Published:** 2024-06-09

**Authors:** Rita T. Michenfelder, Fred Pashley‐Johnson, Viktor Guschin, Laura Delafresnaye, Vinh X. Truong, Hans‐Achim Wagenknecht, Christopher Barner‐Kowollik

**Affiliations:** ^1^ School of Chemistry and Physics Centre for Materials Science Queensland University of Technology (QUT) 2 George St Brisbane QLD 4000 Australia; ^2^ Institute of Organic Chemistry Karlsruhe Institute of Technology (KIT) Fritz‐Haber‐Weg 6 76131 Karlsruhe Germany; ^3^ Institute of Nanotechnology Karlsruhe Institute of Technology (KIT) Hermann‐von‐Helmholtz‐Platz 1 76344 Eggenstein‐Leopoldshafen Germany; ^4^ Polymer Chemistry Research Group Centre of Macromolecular Chemistry (CMaC) and Laboratory of Organic Synthesis Department of Organic and Macromolecular Chemistry Faculty of Sciences Ghent University Krijgslaan 281‐S4 Ghent 9000 Belgium; ^5^ Institute of Sustainability for Chemicals Energy and Environment (ISCE2) Agency for Science, Technology and Research (A*STAR) 1 Pesek Round, Jurong Island Singapore 627833 Republic of Singapore

**Keywords:** photochemical action plots, precision photochemistry, wavelength orthogonal release systems

## Abstract

The wavelength‐by‐wavelength resolved photoreactivity of two photo‐caged carboxylic acids, i. e. 7‐(diethylamino)‐coumarin‐ and 3‐perylene‐modified substrates, is investigated via photochemical action plots. The observed wavelength‐dependent reactivity of the chromophores is contrasted with their absorption profile. The photochemical action plots reveal a remarkable mismatch between the maximum reactivity and the absorbance. Through the action plot data, the study is able to uncover photochemical reactivity maxima at longer and shorter wavelengths, where the molar absorptivity of the chromophores is strongly reduced. Finally, the laser experiments are translated to light emitting diode (LED) irradiation and show efficient visible‐light‐induced release in a near fully wavelength‐orthogonal, sequence‐independent fashion (*λ*_LED1_ = 405 nm, *λ*_LED2_ = 505 nm) with both chromophores in the same reaction solution. The herein pioneered wavelength orthogonal release systems open an avenue for releasing two different molecular cargos with visible light in a fully orthogonal fashion.

## Graphical abstract

The photochemical reactivity of two molecular release systems is demonstrated to be fundamentally disparate from their absorption spectra via photochemical action plots, allowing for the design of pathway‐independent wavelength‐orthogonal photochemical release.

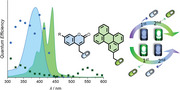

## 1 Introduction

Photochemistry is currently undergoing a precision revolution. While light‐driven chemical processes have been studied for centuries,^[^ ^1^ ^]^ they have commonly been induced by broadly emitting light sources such as mercury and halogen lamps, or just sunlight.^[^ ^2^ ^]^ The use of monochromators in these irradiation sources drastically reduced the excitation power. With the advent of energy‐saving LEDs and monochromatic tunable laser systems, wavelength‐gated processes have become accessible.^[^ ^3^ ^]^ Photochemical processes offer key advantages, most notably their ability to control reactions spatially and temporally,^[^ ^4^ ^]^ which has been exploited in a plethora of applications ranging from curing systems^[^ ^5^ ^]^ and 3D printing^[^ ^6^ ^]^ to inducing chemical processes in living organisms.^[^ ^7^ ^]^ However, inducing chemical reactions with light offers far more opportunities than spatial and temporal control: it allows for the opportunity to control the reaction with the distinct energies available by irradiation with specific colors of light. Controlling chemical reactions via highly energy‐gated processes provides access to four principal photochemical reaction modes that we have recently discussed in terms of their semantic taxonomy.^[^ ^8^ ^]^ Reactions requiring two disparate colors of light to induce a reaction are termed synergistic (when the two colors of light need to be present simultaneously) or cooperative (when the two colors of light can be present sequentially).^[^ ^9^ ^]^ Multiple reactions proceeding independently of each other in the same reaction mixture with distinct colors of light are termed orthogonal, while reactions proceeding with one color of light yet are turned off with another color of light are termed antagonistic.^[^ ^8^, ^10^ ^]^

Such intricate control over photochemical processes can only be achieved if their efficiency is established in a wavelength‐resolved fashion. Over the last decade, our team has developed the photochemical action plot methodology, exploiting tuneable monochromatic laser systems – probing photochemical processes wavelength‐by‐wavelength. Specifically, identical aliquots of a reaction mixture are irradiated with an identical number of photons at each monochromatic wavelength. Subsequently, an analytical technique is employed to determine the conversion of the probed chromophore as a function of irradiation wavelength.^[^ ^8a^ ^]^ The result is a highly wavelength‐resolved image of a specific photochemical process from a defined starting material to a defined reaction product. Using our action plot methodology, we have mapped a wide range of photochemical processes,^[^ ^11^ ^]^ including bond forming^[^ ^12^ ^]^ and cleaving reactions.^[^ ^13^ ^]^ Surprisingly, nearly all of the generated action plots show a marked mismatch between the UV/Vis absorption bands of the chromophore and the maximum reactivity, often finding the maximum reactivity in regions of very low absorptivity. We have recently discussed possible reasons for our findings,^[^ ^14^ ^]^ which have critical consequences for a wide range of applications, including for triggering photochemical processes in living systems, as chromophores that only absorb in the UV range can – in many cases – readily be excited to react in the much more biological benign visible light range.

Photochemical cleavage and release systems, such as *o*‐nitrobenzyl derivatives,^[^ ^15^ ^]^ 7‐(diethylamino)‐coumarin derivatives,^[^ ^16^ ^]^ or 3‐perylene derivatives are an important substrate class and have been employed to, e. g., orthogonally uncage DNA^[^ ^17^ ^]^ or mRNA^[^ ^18^ ^]^ in cells for optical control of transcription or translation processes^[^ ^19^ ^]^ or found application in photolabile hydrogels^[^ ^20^ ^]^ or in controlled light‐responsive drug release.^[^ ^4^, ^21^ ^]^ Multi‐wavelength controlled cleavages have been achieved in an almost wavelength‐selective manner, i. e., longer wavelength activation for the first photo‐induced release, followed by shorter wavelengths for subsequent photo‐uncaging.^[^ ^22^ ^]^ However, achieving full *λ*‐orthogonality^[^ ^23^ ^]^ is challenging and remains largely elusive as it requires exclusively addressing one photo‐active species and reaching high conversions, while the other component remains unaffected, and vice versa.

Indeed, most action plots have been recorded for covalent bond‐forming reactions – most of them showing a mismatch between absorptivity and reactivity, and less attention is placed on photocleavage. To the best of our knowledge, only the photofragmentation of radical initiators for free radical polymerization^[^ ^24^ ^]^ and selected classical photodeprotection reactions have been studied, such as *o*‐nitrobenzenes^[^ ^13^ ^]^ and bimanes.^[^ ^25^ ^]^

**Scheme 1:**
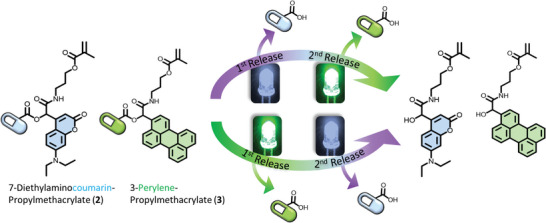
Chemical structures of the chromophores examined in the current study: 7‐diethylaminocoumarin‐propylmethacrylate (**2**) and 3‐perylene‐propylmeth‐acrylate (**3**) alongside their proposed release mechanism. We herein demonstrate that – based on the finding of a photochemical action plot analysis – a wavelength orthogonal photochemical release system can be constructed, where each chromophore can be independently addressed by visible light of a specific color from a one‐pot reaction mixture.

Herein, we pioneer a fully wavelength orthogonal photochemical release system. We use the aforementioned concepts and analytical techniques to develop and characterize a dual carrier system that allows for the fully wavelength‐controlled release of cargo molecules (**Scheme** **1**). We establish photochemical action plots for 7‐(diethylamino)‐coumarin‐ and 3‐perylene‐modified organic compounds (2 and 3), whose photochemical release mechanism is shown in Scheme 1. Our approach to their design is motivated by two factors. First, their absorption spectra are relatively similar but reasonably different, too, to provide a sufficiently high chance that their action plots show disparate reactivity. It is important to note that there is currently no methodology to predict wavelength‐dependent reactivity, as even chromophores with near identical absorption spectra show markedly different wavelength‐resolved photochemical reactivity as recently demonstrated by our team.^[^ ^11b^ ^]^ Second, we included an additional synthetic handle into both release systems to enable their future use.

Here, we introduced a methacrylate functionality that enables the chromophores to potentially be incorporated into a polymeric backbone by radical or ionic polymerization. The action plot methodology we employ herein has been described by us previously^[^ ^8a^ ^]^ and specific modifications for the current chromophores are detailed in Section S4 (Supporting Information).

## 2 Results and Discussion

**Figure 1:**
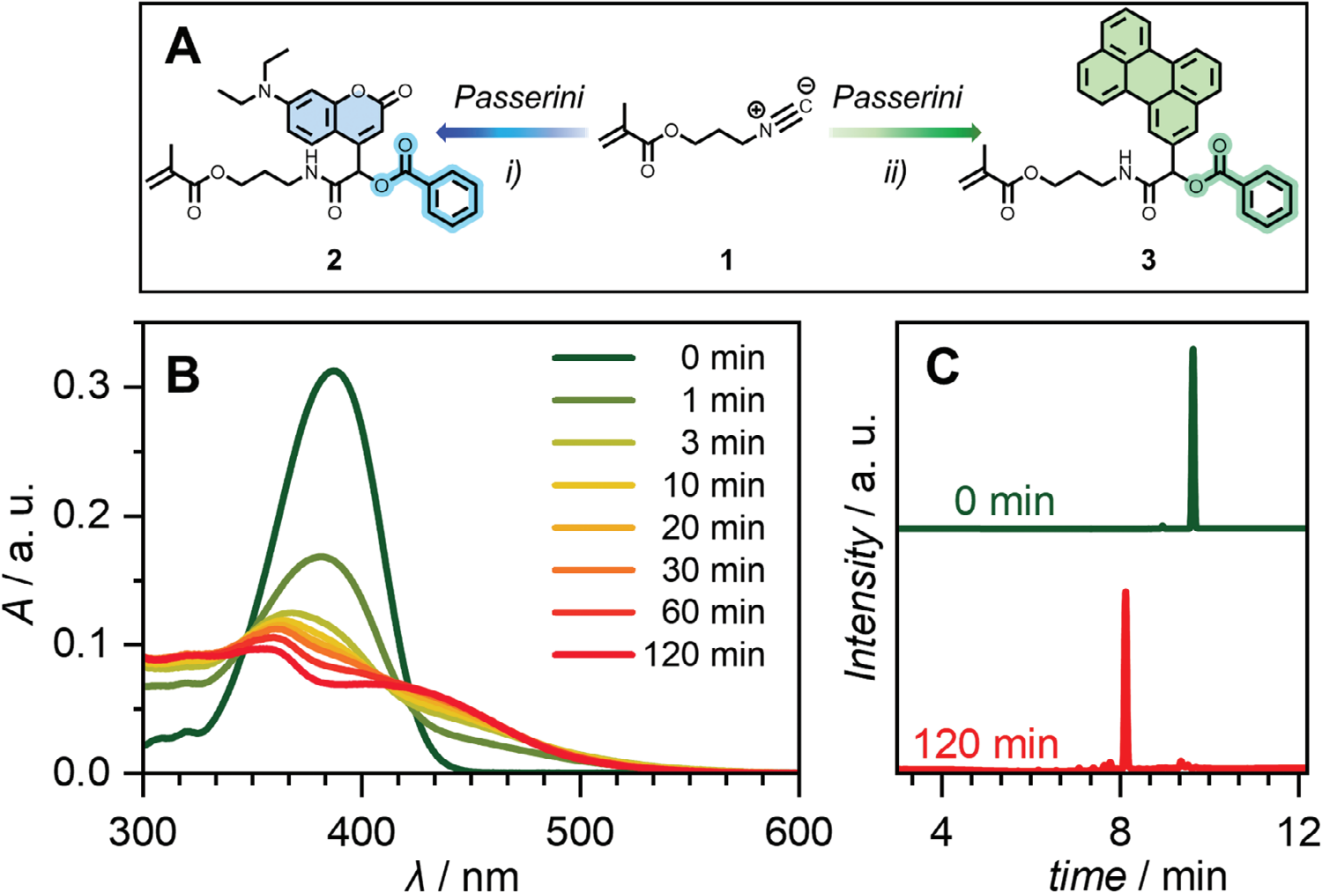
**A)** Synthesis of **2**: i) Benzoic acid, 7‐diethylamine‐1‐methylcoumarin carboxaldehyde, DCM, r. t., 16 h, 92% and **3**: ii) Benzoic acid, 3‐perylenecarboxaldehyde, CHCl_3_, r. t., 16 h, 3%. **B)** UV/Vis absorbance changes of 40 µm **2** over 2 h of irradiation at 20 °C with a 405 nm LED in MeCN. **C)** HPLC results of 40 µm **2** after 2 h of irradiation at 20 °C with a 405 nm LED in MeCN.

Initially, the isocyanide **1** (**Figure** 1A) was synthesized (Figures S1–S7, Supporting Information) as a precursor for the two following Passerini reactions, using aldehyde‐functionalized chromophores (Figure S8, Supporting Information) as well as benzoic acid as model release entity. The coumarin‐modified compound **2** and perylene‐modified compound **3** were isolated in yields up to 92% (Figure 1A). Both molecules were analyzed and characterized via nuclear magnetic resonance (NMR) spectroscopy as well as liquid‐chromatography‐mass‐spectrometry (LC‐MS) (Figure S9–S14, Supporting Information). Subsequently, irradiation experiments with both compounds were performed using a 405 nm LED for the coumarin substrate 2 (*λ*_max_ = 388 nm) and a 445 nm LED for the perylene substrate 3 (*λ*_max_ = 441 nm) to obtain insights regarding the cleavage of the benzoic acid. The reaction was followed by UV/Vis spectroscopy (Figure 1B), and LC‐MS analysis (Figure 1C). Absorbance spectra of **2** (Figure 1B) indicate successful cleavage of the benzoic acid during irradiation, as the absorbance maximum at *λ* = 388 nm decreases.

To further evidence the cleavage mechanism, LC‐MS analysis was performed (Figure 1C). Pre‐irradiation, the chromatogram of **2** shows one significant peak at *t_R_* = 9.67 min (Figure 1C, green line) with *m/z* values corresponding to the Passerini adduct (*m/z*_theo_ = 521.2288, *m/z*_exp_ = 521.2276 for [(2)+H]^+^). During irradiation, this peak decreases in intensity, while a new peak at *t*_R_ = 8.12 min (Figure 1C, red line) arises. Based on the rich literature around the use of (coumarin‐4‐yl)methyl esters as photoprotecting groups,^[^ ^26^ ^]^ we propose that the cleavage mechanism follows similarly to that described by Schade et al.^[^ ^27^ ^]^ (refer to Scheme S6, Supporting Information), eventually yielding the cleaved acid and a hydroxyl‐functionalized partial Passerini adduct. This is evidenced by the LC‐MS data in Figure 1C, as the peak in the red trace has *m/z* values in very good agreement with this structure (*m/z*_theo_ = 417.2026, *m/z*_exp_ = 417.2016 for [(**2‐cleaved**)+H]^+^, Figures S15 and S16, Supporting Information). Furthermore, after the successful scission of the benzoic acid moiety, we anticipated an increase in polarity due to the formation of the hydroxyl functionality, which was confirmed by a shift to shorter elution times in the reverse‐phase high‐performance‐liquid‐chromatography (HPLC) trace (Figure 1C, green line versus red line). Further, we confirmed the successful scission of **2** via NMR spectroscopy. Upon irradiation with an LED centered at 405 nm, the intensity of the coumarin resonances decreases with respect to the internal standard (Figure S36, Supporting Information); concurrently, a resonance at 12.9 ppm increases, relating to the free benzoic acid functionality (Figure S37, Supporting Information). Note that the cleaved structure is not visible in the NMR study – this is attributed to the molecule's poor solubility in the solvent. Successful cleavage was also demonstrated for the perylene‐modified compound **3** to yield the hydroxy‐functionalized Passerini adduct (Figures S17–S19, Supporting Information).

**Figure 2:**
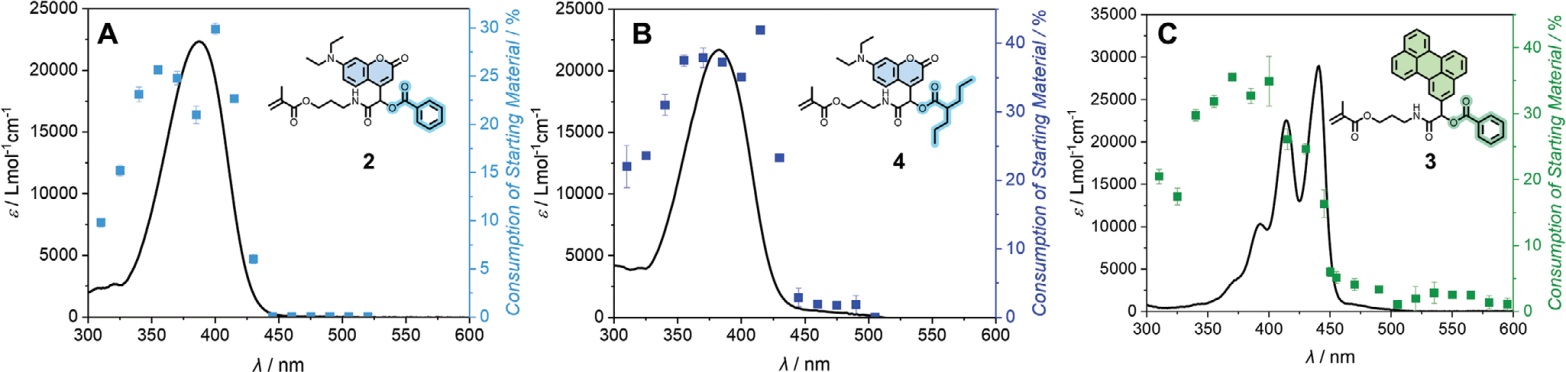
**A)** Molar extinction of **2** overlayed with the action plot consumption of the starting material. For each indicated wavelength, 0.66 µmol photons were deposited into the reaction system (*c* = 147 µm in MeCN). **B)** Molar extinction of **4** overlayed with the action plot consumption of the starting material. For each indicated wavelength 1.93 µmol photons were deposited into the reaction system (*c* = 150 µm in MeCN). **C)** Molar extinction of **3** overlayed with the action plot consumption of the starting material. For each indicated wavelength 7.09 µmol photons were deposited into the reaction system (*c* = 72 µm in MeCN).

Having established our chromophore systems, we subsequently recorded photochemical action plots. We decided to follow the chromophore's photochemical reactivity by mapping the depletion of the starting material, as is usual for photochemical action plots in cleavage systems.^[^ ^8a^ ^]^ The experimental methods for recording photochemical action plots are described in detail in Section S4 (Supporting Information). Briefly, an identical batch of samples is prepared at a suitable concentration, while maintaining sufficient material for characterization. In our case, a concentration of 147 µm (80 µg mL^−1^) was chosen for **2**, whereas a concentration of 72 µm (40 µg mL^−1^) was selected for **3**. At these concentrations, high UV/Vis absorbances without detector saturation were recorded. Each sample is irradiated with an identical number of photons of monochromatic light across the wavelength region of interest. Irradiation is followed by careful quantitative characterization to determine the yield of the reaction. The conversion of **2** was calculated via UV/Vis spectroscopy (refer to Figures S20–S26, Supporting Information for a detailed description of the method), whereas the conversion of **3** was determined using LC‐MS with benzene as an internal standard (refer to Figures S27 and S28, Supporting Information for further details). Once the wavelength‐dependent reactivity has been defined, a wavelength‐dependent reactivity is graphed jointly by overlaying the reactivity with the molar extinction, measured in the reaction solvent, resulting in the chromophores’ action plots (**Figure** **2**). The coumarin derivative's action plot (Figure 2A) clearly shows higher reactivity outside of the absorption maximum (*λ*_max_ = 388 nm) on both sides. Interestingly, there are two reactivity maxima at *λ*_1_ = 350 nm (hypsochromically‐shifted) and *λ*_2_ = 400 nm (bathochromically‐shifted). To evidence that the cleavage is only dependent on the chromophore, but independent of the acid, we synthesized a second coumarin‐modified compound (**4**) using our previously established Passerini protocol. This molecule bears valproic acid as a model release vector (refer to Figures S29–S31, Supporting Information for synthesis). Valproic acid was selected due to its therapeutic uses in epilepsy treatment, bipolar disorder management, and migraine prophylaxis.^[^ ^28^ ^]^ A different operator and laser system were employed to record the action plot of **4** (refer to Figures S32–S34, Supporting Information for a detailed description). The action plot of **4** (Figure 2B) reveals equal reactivity outside of the absorption maximum (*λ*_max_ = 382 nm), in close agreement with the action plot of **2**.

The highest conversions were reached at *λ*_1_ = 370 nm (blue‐shift) and *λ*_2_ = 415 nm (red‐shift), providing strong evidence that the reactivity is only dependent on the employed chromophore, but independent of the acid, user, and employed laser instrument. The perylene monomer (Figure 2C) likewise shows conversion far outside the absorption maxima (*λ*_max1_ = 414 nm, *λ*_max2_ = 441 nm) with the highest conversion occurring at *λ* = 370 nm (hypsochromic shift). Furthermore, we observed conversion at longer wavelengths (*λ* = 450 – 580 nm, bathochromic shift), where the molar absorptivity of the chromophores is extremely low.

**Figure 3:**
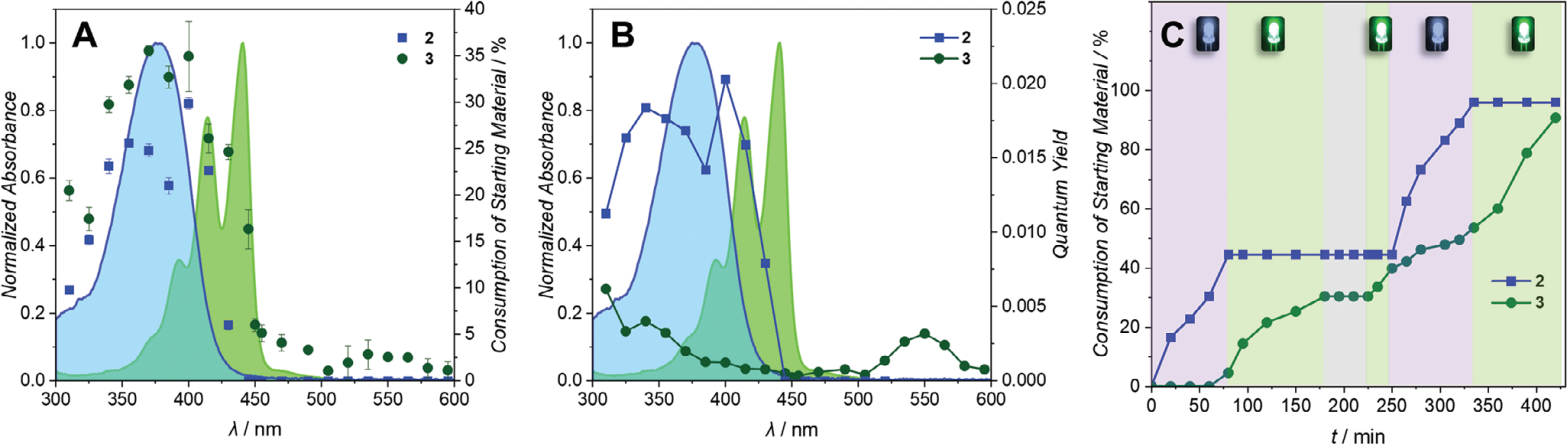
**A)** Dual chromophore action plot for **2** and **3** as well as their molar UV/Vis extinction. The blue line depicts *ε*_Coumarin_ and the green line shows *ε*_Perylene_. The blue squares represent the percentage consumption of the starting material of **2**, while the green dots display the percentage consumption of the starting material of **3. B)** Wavelength‐dependent quantum yield of **2** and **3**. **C)** Consumption of the starting material of **2** and **3** after sequential irradiation with two LEDs (405 nm, 0.2 mW and 505 nm, 40 mW), as well as a dark period. Upon 405 nm irradiation, almost exclusively **2** reacts, while **3** only reacts after exposure to 505 nm light. Tabulated data for the experiment can be found in Table S7 (Supporting Information).

By overlaying both action plots in a dual chromophore graph (**Figure** **3**A), we aimed to determine a reactivity window, where both chromophores can be independently activated. Therefore, we can achieve wavelength‐orthogonal cleavage of the acids from a one‐pot mixture containing both chromophores as starting material. As both compounds show reactivity in the shorter wavelength region, there appears to be no immediate avenue to orthogonality by choice of color alone. Nevertheless, at λ ≥ 450 nm, only the perylene‐modified compound **3** reacts, whereas the coumarin‐modified substrate **2** does not cleave. However, **2** reacts faster than **3** as confirmed by the number of photons that were employed to record the action plots. To obtain close to 25% conversion with **2** , 0.66 µmol of photons (4.00 × 10^17^ photons) were deposited into the system, whereas for **3**, 7.09 µmol of photons (4.27 × 10^18^ photons) were required to obtain 25% consumption of the starting material (refer to Figures S25 and S27, Supporting Information for detailed description of the reaction kinetics). To further assess the orthogonality of the two photochemical reactions, we calculated the wavelength‐dependent quantum yield (Figure 3B) for each point on the action plot using the methodology described in Section S8 (Supporting Information). The data highlights the difference in reactivity between the two chromophores and allow for the selection of irradiation conditions to facilitate near‐perfect sequence‐independent orthogonal release. Utilizing the difference in quantum yields between **2** and **3**, we can use low‐intensity 405 nm light to exclusively trigger the release of **2**, whilst higher intensity 505 nm light will function as the trigger for photochemically decaying **3**. Further, based on the calculation of the number of photons, two‐photon‐induced processes can be ruled out (refer to Section S8, Supporting Information). We note that the identification of pathway‐independent wavelength orthogonality would not have been possible by merely inspecting the absorption spectra of the two chromophores.

Having established the wavelengths with the highest selectivity (405 and 505 nm) and considering the rates at which each chromophore responds to these wavelengths, we established a near pathway independent *λ*‐orthogonal cleavage system in a one‐pot reaction (Figure 3C). The irradiation sequence includes both possible pathways (high energy to low energy and low energy to high energy) as well as a dark period. A 0.20 mW LED centered at 405 nm was used to cleave the acid from the coumarin‐modified compound **2**, whereas a 40 mW LED, centered at 505 nm, was employed to perform cleavage of the perylene‐modified compound **3** (refer to Figure S35, Supporting Information for emission spectra of the LEDs and experimental setup, and Figures S39 and S40, Supporting Information for single‐color irradiations). We initially tested the high energy to low energy sequence, starting with the 405 nm LED. Due to the low output power of the 405 nm LED, significantly fewer photons per unit of time are deposited into the reaction system. Samples were taken in 20‐min intervals and only **2** released the acid. After a total of 80 min, we found 45% conversion of the coumarin‐modified compound **2**, while only 5% conversion of the perylene‐modified compound **3** was observed. After switching to the 505 nm LED, samples were taken every 30 min due to the longer reaction times and larger number of photons required for the perylene cleavage. After a total irradiation time of 90 min, 30% cleavage of **3** was recorded, whereas the amount of **2** remained unaffected. In the following 45 min of dark period, no increase in conversion in either of the compounds was observed. Subsequently, the low energy to high energy irradiation sequence was executed, starting with 505 nm LED irradiation. During the 60 min green light irradiation, **3** continued to react and finally afforded 40% conversion, while **2** remained constant. After switching to 405 nm LED irradiation for 75 min again, 95% of **2** was consumed, yet also 13% more of **3** was cleaved off due to slower but still existing reactivity of **3** in the lower wavelength range. It is not likely that the decreased pH of the solution upon irradiation (due to the photorelease of benzoic acid) plays a role in the reactivity of **3** in the second irradiation window of 405 nm. Figure S38 (Supporting Information) shows that with an extreme excess of benzoic acid (53 mm) and therefore increased acidity of the solution, the reactions progress slightly slower. Finally, irradiation was reverted back to 505 nm, while taking a sample every 30 min. A final conversion of 90% was obtained for **3**, whereas the amount of **2** remained constant.

## 3 Conclusion

We report the wavelength‐resolved photocleavage of 7‐(diethylamino)‐coumarin‐modified compound **2** and 3‐perylene‐modified compound **3** as potential wavelength orthogonal delivery system and contrast the observed wavelength‐dependent reactivity with the extinction of the chromophores. After initial LED experiments to understand the photochemical cleavage process, photochemical action plots were recorded for both chromophores using a tuneable ns‐laser system to determine the most suitable wavelengths for *λ*‐orthogonality. Both molecules revealed high photoreactivity disparate to their respective absorbance maxima. Although both compounds showed reactivity in the shorter wavelength region, near pathway independent λ‐orthogonality was established underpinned by the slower reaction kinetics of the perylene compound compared to the coumarin compound under the same photon flux. We employed two broad LEDs (centered at 405 nm and 505 nm) for sequential irradiation of both compounds in one reaction mixture, demonstrating the individual addressable nature of the systems. We submit that the inspection of UV/Vis absorption alone is not sufficient for the design of wavelength orthogonal reaction systems. Such advanced photochemical reaction processes can only be established based on wavelength‐resolved photochemical action plots, as photochemical reactivity and absorptivity can be strongly mismatched. Our findings with regard to the two employed chromophores offer critical potential for photochemical delivery applications with multiple release profiles.

## Conflict of Interest

The authors declare no conflict of interest.

## Author Contributions

R.T.M. and F.P.‐J. contributed equally to this work. R.T.M. conducted the syntheses and photochemical experiments and recorded the action plots. F.P.‐J. conducted the LC‐MS experiments, developed the solver function in Excel to determine the conversion of the coumarin monomer, and calculated the wavelength‐dependent quantum yields. V.G. assisted with the syntheses as well as the recording of the action plots. C.B.‐K., H.‐A.W., and V.X.T. conceptualized the study and determined the research direction. C.B.‐K. and H.‐A.W. acquired funding. L.D., V.X.T., H.‐A.W., and C.B.‐K. supervised the research project. All authors discussed the data and co‐edited the manuscript.

## Supporting information

Supporting Information

## Data Availability

The data that support the findings of this study are available from the corresponding author upon reasonable request.
