# Supplementary material for: Photochemical Action Plots Map Orthogonal Reactivity in Photochemical Release Systems
Source: Adv Sci (Weinh). 2024 Jun 9;11(29):2402011. doi: 10.1002/advs.202402011 (PMC11304248; doi:10.1002/advs.202402011)
Supplement: Supplementary file 1 — Supporting Information [file ADVS-11-2402011-s001.pdf]

ADVANCED SCIENCE
Open Access

Supporting Information

for *Adv. Sci.*, DOI 10.1002/advs.202402011

Photochemical Action Plots Map Orthogonal Reactivity in Photochemical Release Systems

Rita T. Michenfelder, Fred Pashley-Johnson, Viktor Guschin, Laura Delafresnaye, Vinh X. Truong, Hans-Achim Wagenknecht* and Christopher Barner-Kowollik**

Photochemical Action Plots Mapp Orthogonal Reactivity in Photochemical Release Systems

Rita T. Michenfelder‡, Fred Pashley-Johnson‡, Viktor Guschin, Laura Delafresnaye, Vinh X. Truong*, Hans-Achim Wagenknecht* and Christopher Barner-Kowollik*

Abstract:

The wavelength-by-wavelength resolved photoreactivity of two photo-caged carboxylic acids , i. e. 7-(diethylamino)-coumarin- and 3-perylene-modified substrates, is investigated via photochemical action plots. The observed wavelength-dependent reactivity of the chromophores is contrasted with their absorption profile. The photochemical action plots reveal a remarkable mismatch between the maximum reactivity and the absorbance. Through the action plot data, the study is able to uncover photochemical reactivity maxima at longer and shorter wavelengths, where the molar absorptivity of the chromophores is strongly reduced. Finally, the laser experiments are translated to light emitting diode (LED) irradiation and show efficient visible-light-induced release in a near fully wavelength-orthogonal, sequence-independent fashion (λLED1 = 405 nm, λLED2 = 505 nm) with both chromophores in the same reaction solution. The herein pioneered wavelength orthogonal release systems open an avenue for releasing two different molecular cargos with visible light in a fully orthogonal fashion.

Table of Contents

1 Materials and Methods 3
2 Synthetic Procedures 5
2.1 Synthesis of Compound 1 5
2.2 Synthesis of Compound 2 14
2.3 Synthesis of Compound 3 18
3 Additional Data and Spectra 23
3.1 Compound 2 23
3.2 Compound 3 25
4 Tuneable Laser Experiments 28
4.1 Laser Set Up 28
4.2 Control over Incident Photon Number 29
4.3 Kinetic Measurements and Action Plot of Compound 2 30
4.3.1 Results of the Kinetic Experiments of Compound **2** 30
4.3.2 Action Plot of Compound **2** 33
4.4 Kinetic Measurements and Action Plot of Compound 3 36
4.4.1 Results of the Kinetic Experiments of Compound **3** 36
4.4.2 Action Plot of Compound **3** 37
5 Compound 4 39
5.1 Synthesis of Compound 4 39
5.2 Analysis of Compound 4 44
5.2.1 Results of the Kinetic Experiments of Compound **4** 45
5.2.2 Action Plot of Compound **4** 46
6 LED Experiments 48
6.1 LED Emission Spectra and Irradiation Set Up 48
6.2 Results of the Sequential LED Experiment 49
7 Proposed Mechanism of Photocleavage 50
8 Calculation of Quantum Yield and Two-Photon Reactivity 51
9 NMR Study of Irradiation of 2 52
10 Control Irradiation Experiments 53
11 References 55

1 Materials and Methods

All reactions were either performed under a dry argon environment or under inert atmosphere, as detailed for each reaction in section 2 (Synthetic Procedures). All solvents and reagents were commercially purchased at ABCR, ACROS Organics, ALFA Aesar, Carl Roth, Chem Supply, CombiBlocks, Merck, Sigma Aldrich, ThermoFisher Scientific and VWR. Unless stated otherwise, all chemicals were used as received without further purification. Anhydrous solvents were purchased at ACROS Organics and stored under argon. HPLC grade solvents were acquired at Fisher Scientific, deuterated solvents for NMR spectroscopy were purchased at Eurisotop. Water was deionized using a Merck Millipore-Q8. For reactions under inert conditions, flasks and other glassware were heated with a heat gun and dried in high vacuum, followed by flooding with argon (99.999 % purity). Room temperature refers to ambient temperature (20-22 °C). The silica gel for flash chromatography (pore size 60 Å, particle size 40-63 µM) was purchased at Sigma Aldrich. The crude product was either dissolved in the solvent system or adsorbed onto silica before purification. Pressure was applied to the column using an air pump. Reactions were monitored by Thin Layer Chromatography (TLC) using silica gel 60 F_{254} coated aluminum plates by Merck. For detection, λ = 254 nm (fluorescence deletion) or λ = 366 nm (fluorescence excitation) was used. For staining, 5 % H_2SO_4 in MeOH, $KMnO_4$ solution (1.50 $KMnO_4$, 10.0 g K_2CO_3, 1.85 mL 10 % NaOH and 200 mL H_2O) or 0.5 % Ninhydrin in Butan-1-ol (spray reagent) were used.

Spectroscopy

UV/Vis spectroscopy

UV/Vis absorbance spectra were recorded on a Shimadzu UV-2700 spectrophotometer equipped with a CPS-100 electronic temperature control cell positioner. Samples were prepared in MeCN and measured using 1 cm quartz glass cuvettes (Starna) with either a sample volume of 500 µL or 1 mL at 25 °C. Molar absorption coefficient were obtained by measuring spectra at various concentrations and applying Beer-Lambert's law to calculate a linear fit, $A = \varepsilon c d$.

NMR spectroscopy

NMR spectroscopic data were either recorded on a Bruker System 600 Ascend LH, equipped with a BBO-Probe (5mm) with z-gradient (^{1}H: 600.13 MHz, ^{13}C: 150.90 MHz) or on a Bruker Avance 400 MHz spectrometer (^{1}H: 400.13 MHz, ^{13}C: 100.90 MHz) at ambient temperature. 10 mg of compound was dissolved in 400 µL deuterated solvent. The chemical shifts are reported in δ units, parts per million (ppm) downfield from TMS. Coupling constants (J) are given in Hertz (Hz) and multiplicities are abbreviated as following: s (singlet), d (doublet), t (triplet), dd (doublet of doublets), td (triplet of doublets), ddd (doublet of doublet of doublets), tt (triplet of triplets), p (pentet), m (multiplet), b (broad). Various 2D technique experiments were used to establish the structures and to assign the signals.

Mass spectrometry

LC-MS

QUT:

Liquid-chromatography coupled mass spectrometry (LCMS) measurements were performed on an Ultimate 3000 UHPLC System (Dioney, Sunnyvale, CA, USA) consisting of a pump (LPG 3400SZ), autosampler (WPS 3000TSL) and a temperature-controlled column compartment (TCC 3000). Separation was performed on a C_{18} column (Phenomenex Luna 5 µm, 100 Å, 250 x 2.0 mm), operating at 40 °C. A Gradient of MeCN : H_2O 5:95 to 100:0 (v/v) during 7 min at a flow rate of 1.0 mL min^{-1} was applied. The flow was split in a 9:1 ratio, where 90 % of the eluent was directed through a DAD UV-Detector (VWD 3400, Dionex) and 10 % was injected into the electrospray source. Spectra were recorded on a LTQ Orbitrap Elite mass spectrometer (Thermo Fisher Scientific, San Jose, CA, USA) equipped with a HESI II probe. The instrument was calibrated in the m/z range between 74-1822 using premixed calibration solutions (Thermo Fisher Scientific). A constant spray voltage of 3.5 kV was applied, and the capillary temperature set to 300 °C. Samples were prepared in a concentration of 0.5 mg mL^{-1} in MeCN and filtered through 0.22 µm PTFE membrane filters prior to injection.

KIT:
LCMS measurements were performed on an Agilent 1260 Infinity II system consisting of a quarternary pump (GB7111B), autosampler (G7129A, 100 µL sample loop), a temperature-controlled column oven (G7114A) and a variable UV-Vis detector (G7114A, VWD, flow cell G7114A 018, d = 10 mm, V = 14 µL). Separation was performed on a C18 HPLC column (Agilent Poroshell 120 EC-C18 4.6x100mm, 2.7 µm) operating at 40 °C. A gradient of MeCN : H_2O 10:90 – 80:20 (v/v) (additive 10 mmol L^{-1} $NH_4CH_3CO_2$) at a flow rate of 1 mL min^{-1} during 15 min was used as the eluting solvent. The flow was directed into an Agilent MSD (G613BA, AP-ESI ion source). The instrument was calibrated in the *m*/*z* range 118-2121 in positive mode and 113-2233 in the negative mode, using a premixed calibration solution (Agilent). The following parameters were used: spray chamber flow: 12 L min^{-1}, drying temperature: 350 K, Capillary Voltage: 3000 V, Fragmentor Voltage: 100 V.

2 Synthetic Procedures

2.1 Synthesis of Compound 1

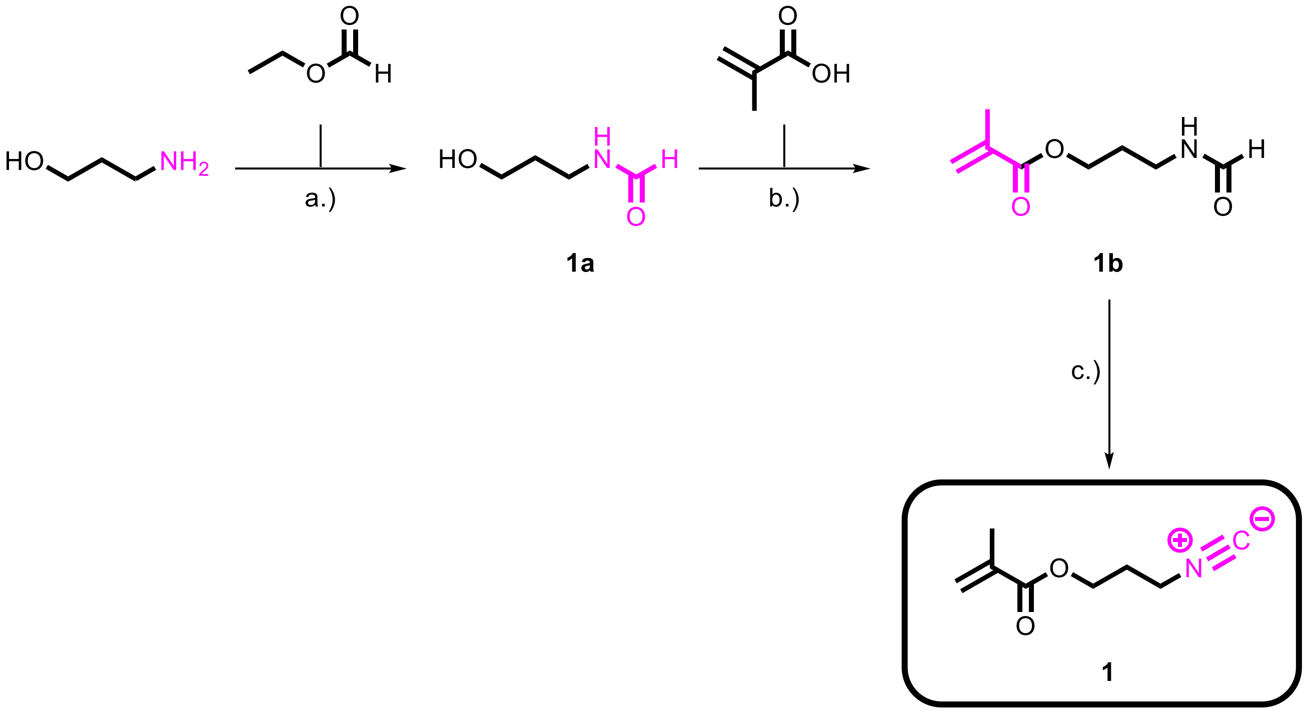

Scheme S1: Synthesis of isocyanide **1**: a) MeOH, 50 °C, 2 h, quant.; b) EDC-HCl, DMAP, r. t., 16 h, 17%; c) Pyridine, p-TsCl, DCM, 2 h, r. t., 2 h, 72%.

Compound 1a

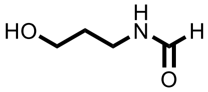

$C_4H_9NO_2$
103.1210 g mol^{-1}

1.00 mL 3-aminopropan-1-ol (1.00 g, 13.3 mmol, 1.00 equiv.) was dissolved in 10 mL MeOH and cooled with an ice bath. 1.08 mL ethylformate (985 mg, 13.3 mmol, 1.00 equiv.) was added portion-wise to the stirred solution over a period of 15 min. Subsequently, the solution was removed from the ice bath and heated at 50 °C for 2 h. The solvent was removed under reduced pressure to afford 1.35 g (13.2 mmol, 99%) of the desired product as a colorless oil. This compound decomposes upon storage and was immediately used in the next step.

R*f* (DCM / MeOH 10:1) = 0.23 – The product was stained using $KMnO_4$-solution.

^{1}H-NMR (600 MHz, DMSO-*d*$_6$): *δ* (ppm) = 7.98-7.95 (m, 1H, C*H*O), 4.29 (bs, 1H, O*H*), 3.41 (t, *J* = 6.3 Hz, 2H, C*H*$_2$), 3.12 (td, *J* = 7.1, 5.6 Hz, 2H, C*H*$_2$), 1.55 (p, *J* = 6.6 Hz, 2 H, C*H*$_2$).

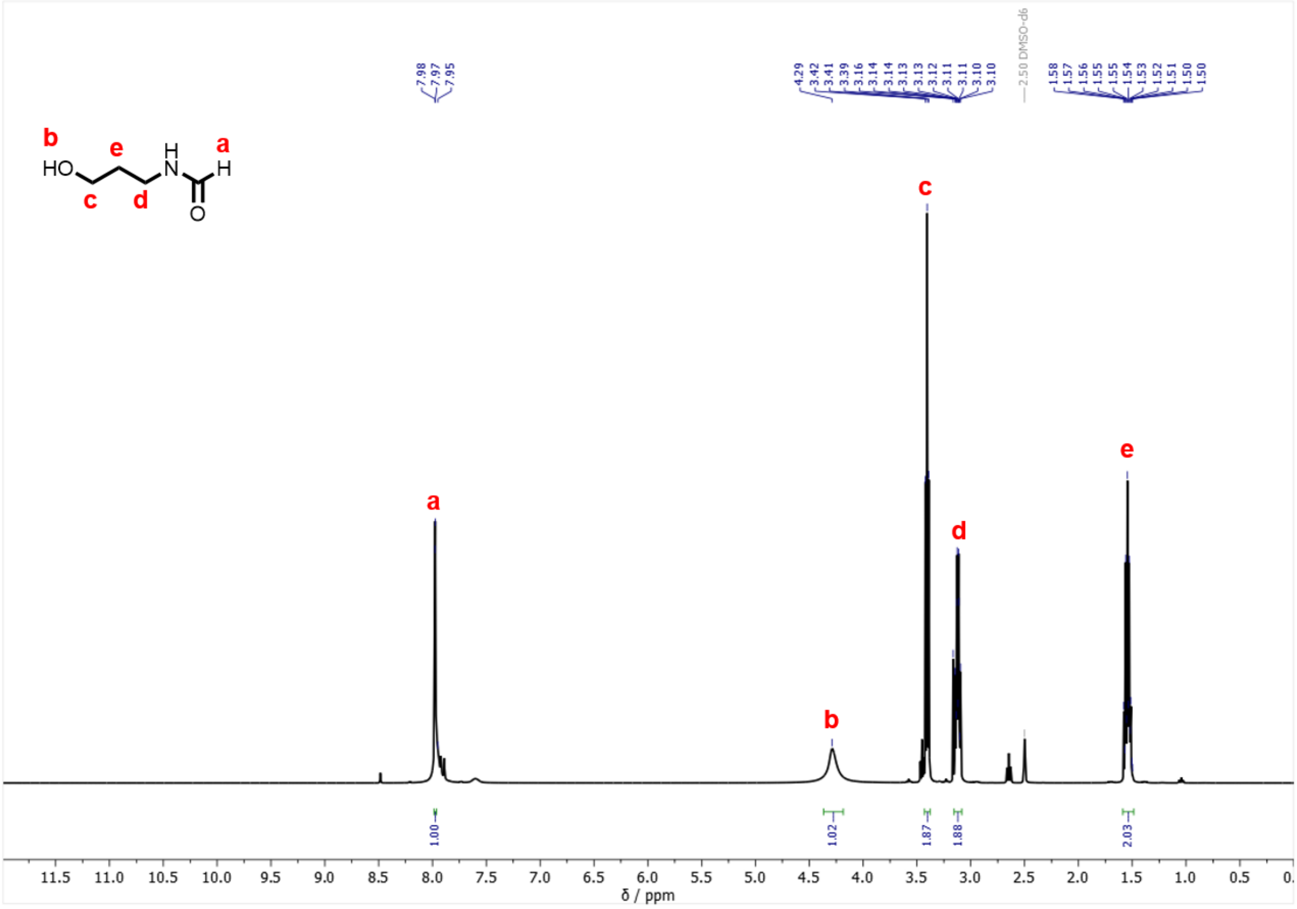

Figure S1: ^{1}H-NMR spectrum of compound **1a** (DMSO-*d*$_6$, 600 MHz).

^{13}C-NMR (151 MHz, DMSO-d_6): δ (ppm) = 161.2 (s, 1C, *C*HO), 58.5 (s, 1C, *C*H_2), 34.5 (s, 1C, *C*H_2), 32.3 (s, 1C, *C*H_2).

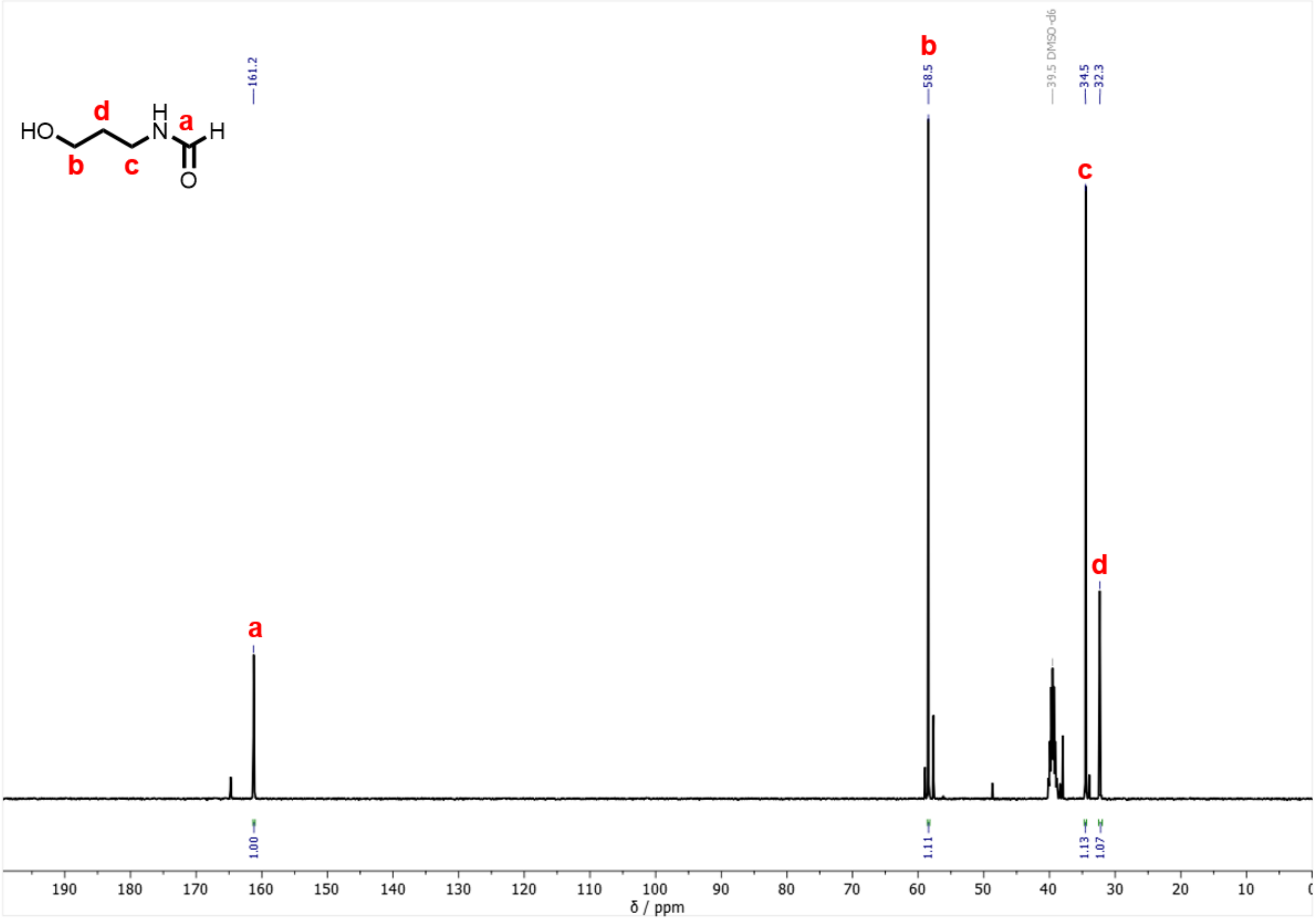

Figure S2: ^{13}C-NMR spectrum of compound **1a** (DMSO-d_6, 151 MHz).

Compound 1b

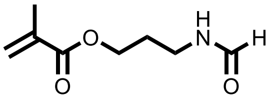

$C_8H_{13}NO_3$
171.1960 g mol^{-1}

3.80 g EDC-HCl (19.9 mmol, 1.50 equiv.) and 1.50 mL methacrylic acid (1.39 g, 13.3 mmol, 1.00 equiv.) were dissolved in 40 mL dry DCM under argon atmosphere and stirred for 30 min at ambient temperature. 1.35 g **1a** (13.3 mmol, 1.00 equiv.) and 2.43 g DMAP (19.9 mmol, 1.50 equiv.) was added, and the reaction mixture stirred for 16 h at ambient temperature. 20 mL DCM was added and the organic phase was extracted with brine (4 x 30 mL) and water (4 x 30 mL). The organic phase was dried over Na_2SO_4 and the solvent removed under reduced pressure. Column chromatography (DCM / MeOH 10:1) delivered 426 mg product (2.50 mmol, 19%) as a colorless oil.

$\mathbf{R}_f$ (5% MeOH in DCM) = 0.33 – The product was stained using $KMnO_4$-solution.

^{1}**H-NMR** (600 MHz, DMSO-d_6): δ (ppm) = 8.05 (bs, 1H, N*H*), 8.01 (s, 1H, C*H*O), 6.03 (s, 1H, C*H*), 5.67 (t, J = 1.6 Hz, 1H, C*H*), 4.10 (t, J = 6.4 Hz, 2H, CH_2), 3.17 (q, J = 6.5 Hz, 2H, CH_2), 1.88 (s, 3H, CH_3), 1.77 (p, J = 6.6 Hz, 2H, CH_2).

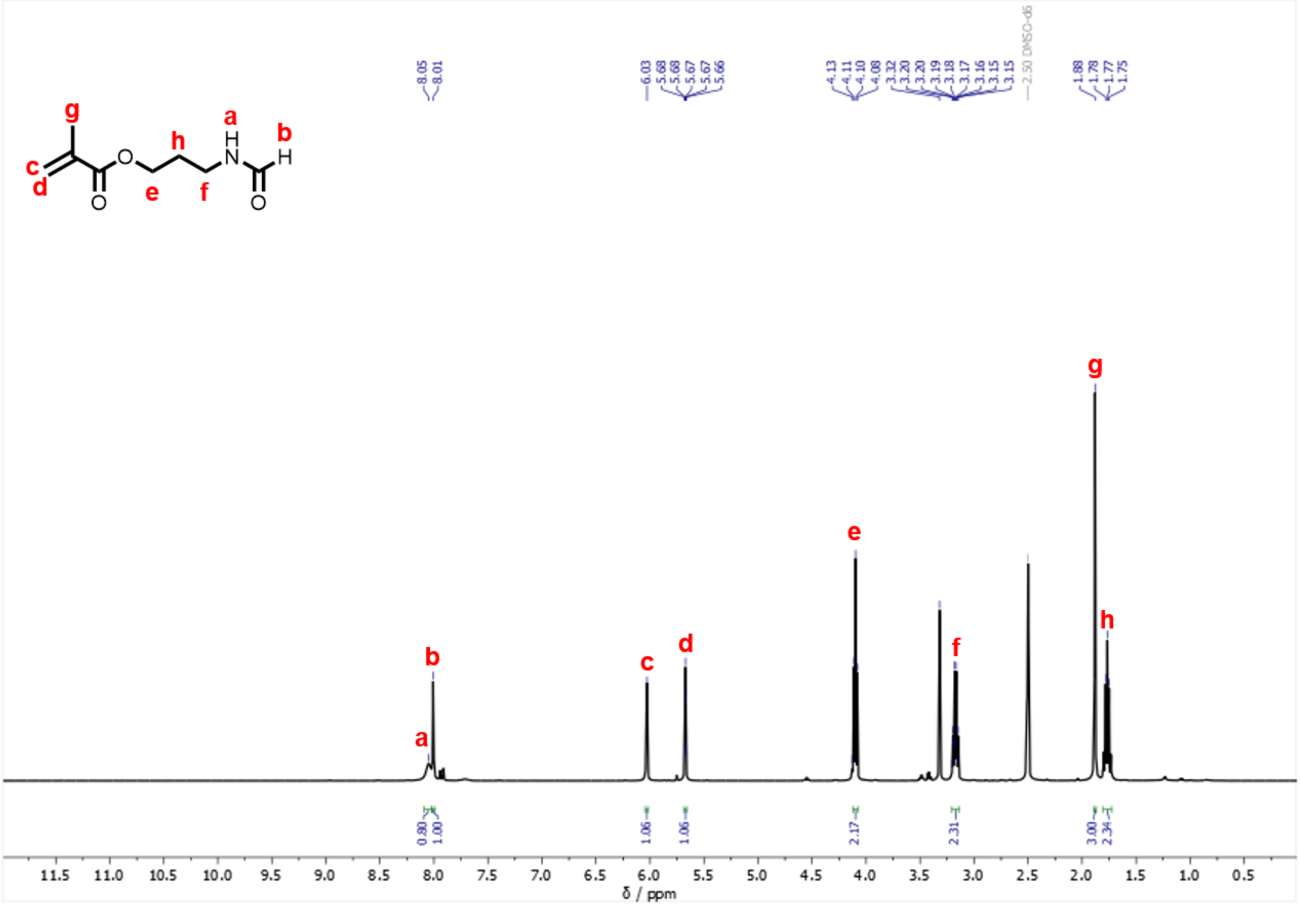

Figure S3: ^{1}H-NMR spectrum of compound **1b** (DMSO-d_6, 600 MHz).

^{13}C-NMR (151 MHz, DMSO-d_6): δ (ppm) = 166.5 (s, 1C, *C*HO), 161.1 (s, 1C, *C*HO), 135.9 (s, 1C, *C*H_2), 125.6 (s, 1C, *C*H_2), 62.0 (s, 1C, *C*H_2), 34.0 (s, 1C, *C*H_2), 28.2 (s, 1C, *C*H_2), 17.9 (s, 1C, *C*H_3).

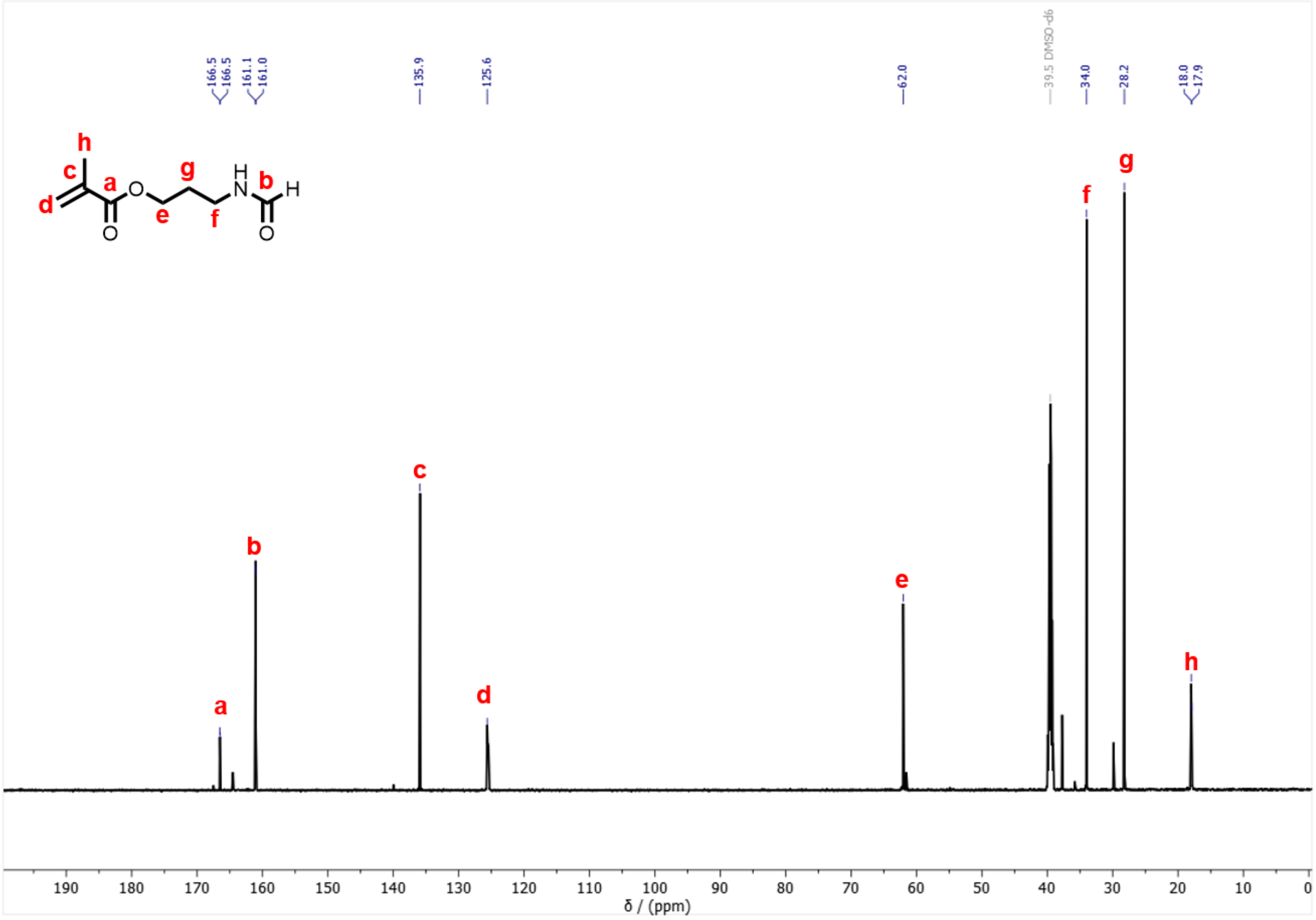

Figure S4: ^{13}C-NMR spectrum of compound **1b** (DMSO-d_6, 151 MHz).

LC-MS:

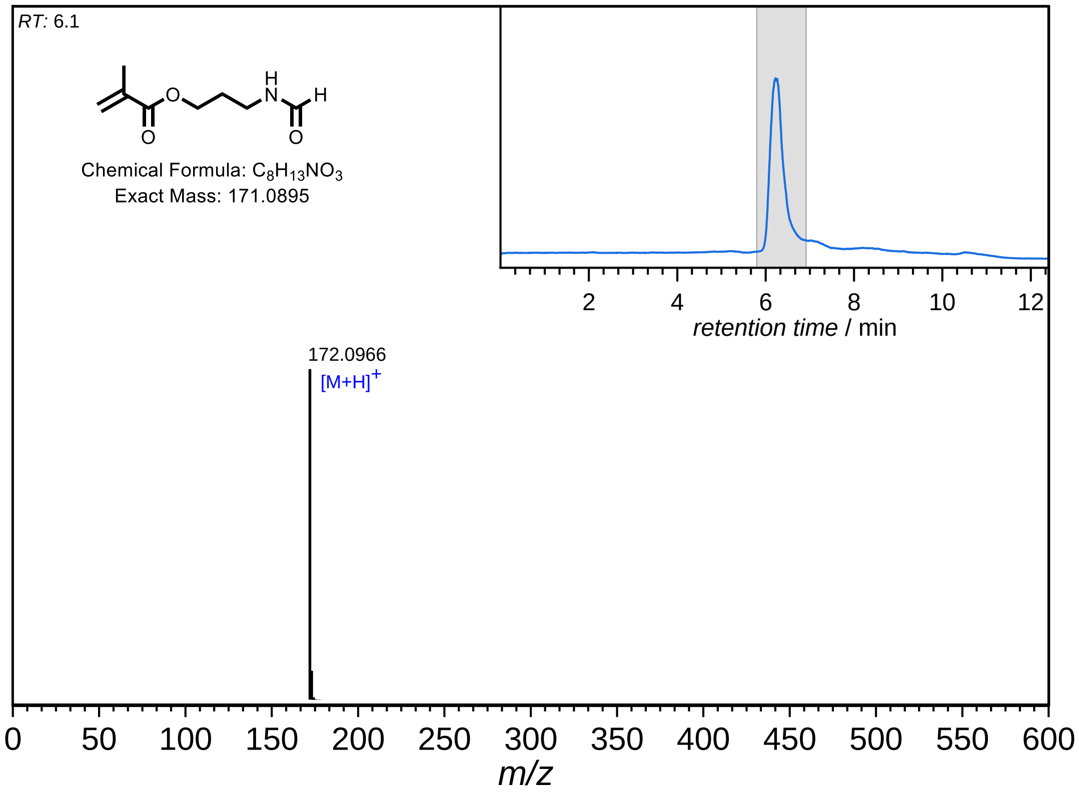

Figure S5: LC-MS analysis of compound **1b**: m/z_{theo} = 171.0895 [$C_8H_{13}NO_3$], m/z_{exp} = 172.0966 $[M+H]^+$.

Compound 1

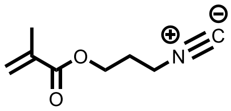

$C_8H_{11}NO_2$
153.1810 g mol^{-1}

365 mg **1b** (2.13 mmol, 1.00 equiv.) was dissolved in 15 mL DCM and 515 µL pyridine (505 mg, 1.75 mmol, 3.00 equiv.) was added. Subsequently, 167 mg *p*-TsCl (876 µmol, 1.50 equiv.) was added under cooling with an ice bath. Cooling was removed and the reaction mixture stirred until full conversion (monitored via TLC, average reaction time of 2 h) was observed. Subsequently, 10 mL aqueous Na_2SO_4-solution (20 wt%) was added and the biphasic mixture stirred for another 30 min. 10 mL water and 10 mL DCM were added and the organic phase was separated. The aqueous phase was extracted with DCM (3 x 10 mL), the organic extracts were combined and washed with water (3 x 10 mL) and brine (2 x 10 mL). The organic extract was dried over Na_2SO_4, filtered and the solvent was removed under reduced pressure. The crude product was purified via column chromatography (1% MeOH in DCM) to obtain 235 mg of the isocyanide (1.53 mmol, 72%) as a pale-yellow oil with a distinct smell.

R*f* (DCM) = 0.65 – The product was stained using $KMnO_4$-solution.

^{1}H-NMR (600 MHz, DMSO-d_6): δ (ppm) = 6.07 (dq, J = 1.9, 1.0 Hz, 1H, C*H*), 5.69 (q, J = 1.6 Hz, 1H, C*H*), 4.18 (t, J = 6.1 Hz, 2H, CH_2), 3.61 (ddd, J = 8.6, 5.2 Hz, 2.0 Hz, 2H, CH_2), 1.97 (ttd, J = 11.0, 6.3, 5.5 Hz, 2.4 Hz, 2H, CH_2), 1.89 (dd, J = 1.6, 1.0 Hz, 3H, CH_3).

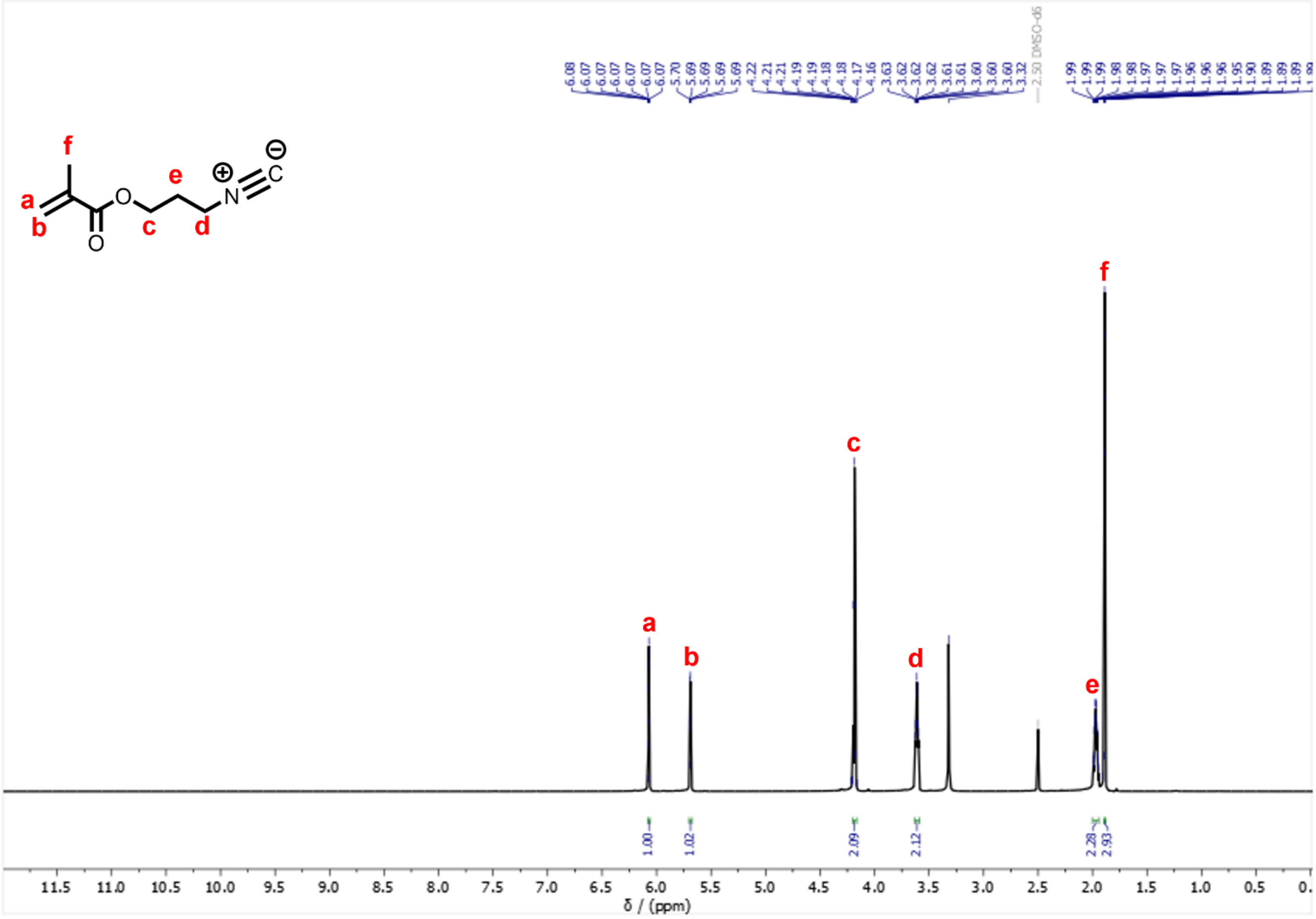

Figure S6: ^{1}H-NMR spectrum of compound **1** (DMSO-d_6, 600 MHz).

^{13}C-NMR (151 MHz, DMSO-d_6): δ (ppm) = 166.4 (s, 1C, *C*N), 156.1 (s, 1C, *C*HO), 135.7 (s, 1C, *C*H_2), 126.0 (s, 1C, *C*H_2), 61.2 (s, 1C, *C*H_2), 38.5 (s, 1C, *C*H_2), 27.7 (s, 1C, *C*H_2), 18.0 (s, 1C, *C*H_3).

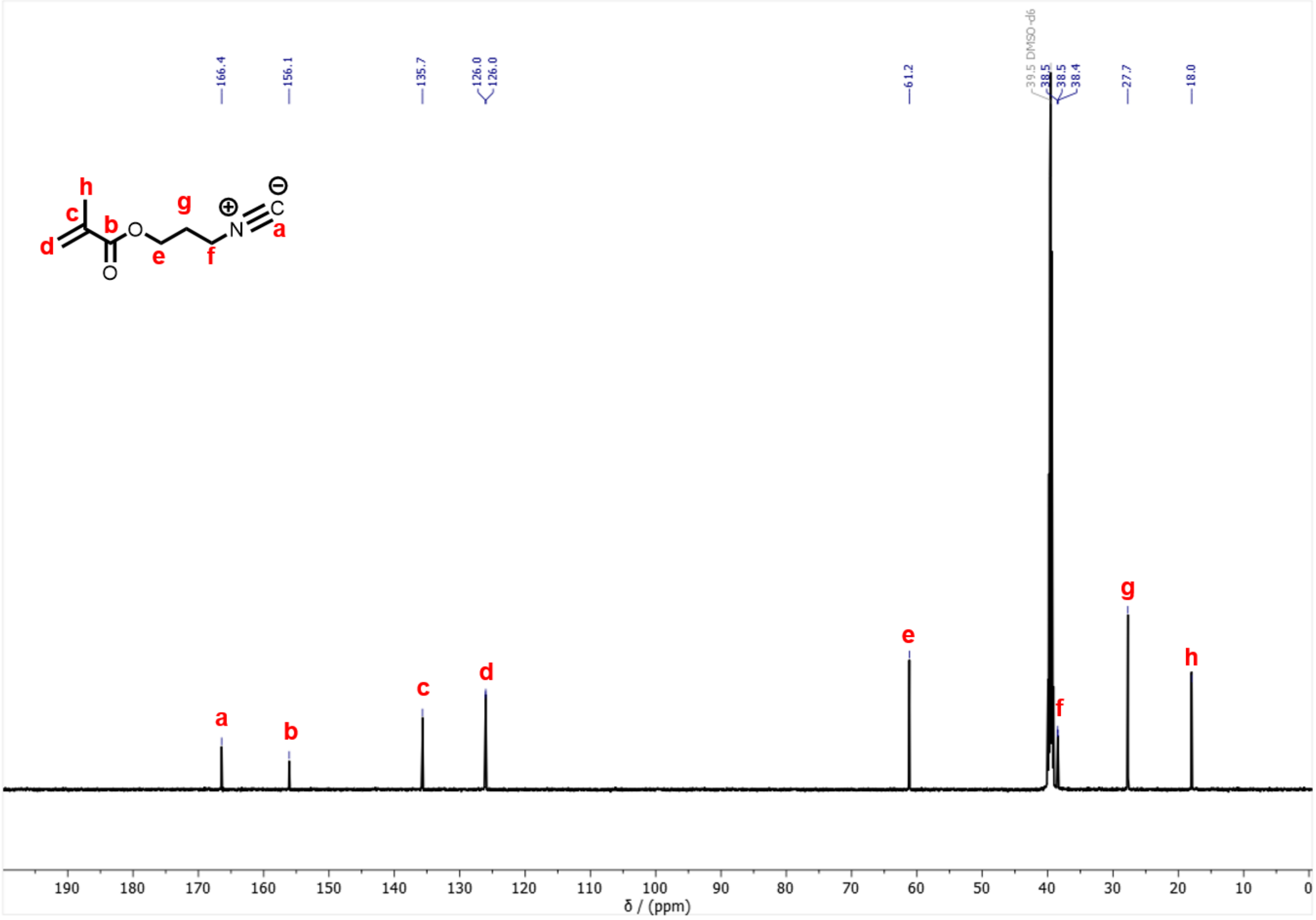

Figure S7: ^{13}C-NMR spectrum of compound **1** (DMSO-d_6, 151 MHz).

Compound 1c

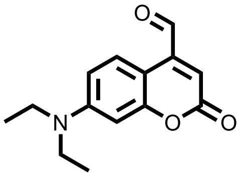

$C_{14}H_{15}NO_3$
245.2780 g mol^{-1}

2.30 g 7-diethylamine-1-methylcoumarin (10.0 mmol, 1.00 equiv.) and 1.66 g SeO_2 (15.0 mmol, 1.50 equiv.) was dissolved in 60 mL Xylene and the mixture heated at 130 °C under refluxing conditions. The solution was filtered when hot and the filtrate was concentrated in vacuo. The crude product was purified via column chromatography on silica gel running with *n*-Hex / EtOAc (3:1) to give 1.39 g of the product (5.67 mmol, 57%) as a red solid.

R*f* (*n*-Hex / EtOAc 3:1) = 0.42.

^{1}H-NMR (400 MHz, DMSO-d_6): δ (ppm) = 10.07 (s, 1H, C*H*O), 8.19 (d, J = 9.2 Hz, 1H, Ar-*H*), 7.88 (d, J = 9.0 Hz, 1H, Ar-*H*), 6.77-5.58 (m, 1H, Ar-*H*), 6.49 (d, J = 2.5 Hz, 1H, Ar-*H*), 5.92 (d, J = 1.4 Hz, 1H, Ar-*H*), 3.46-3.39 (m, 4H, 2x CH_2),1.12 (td, J = 1.2 Hz, 6H, 2x CH_3).

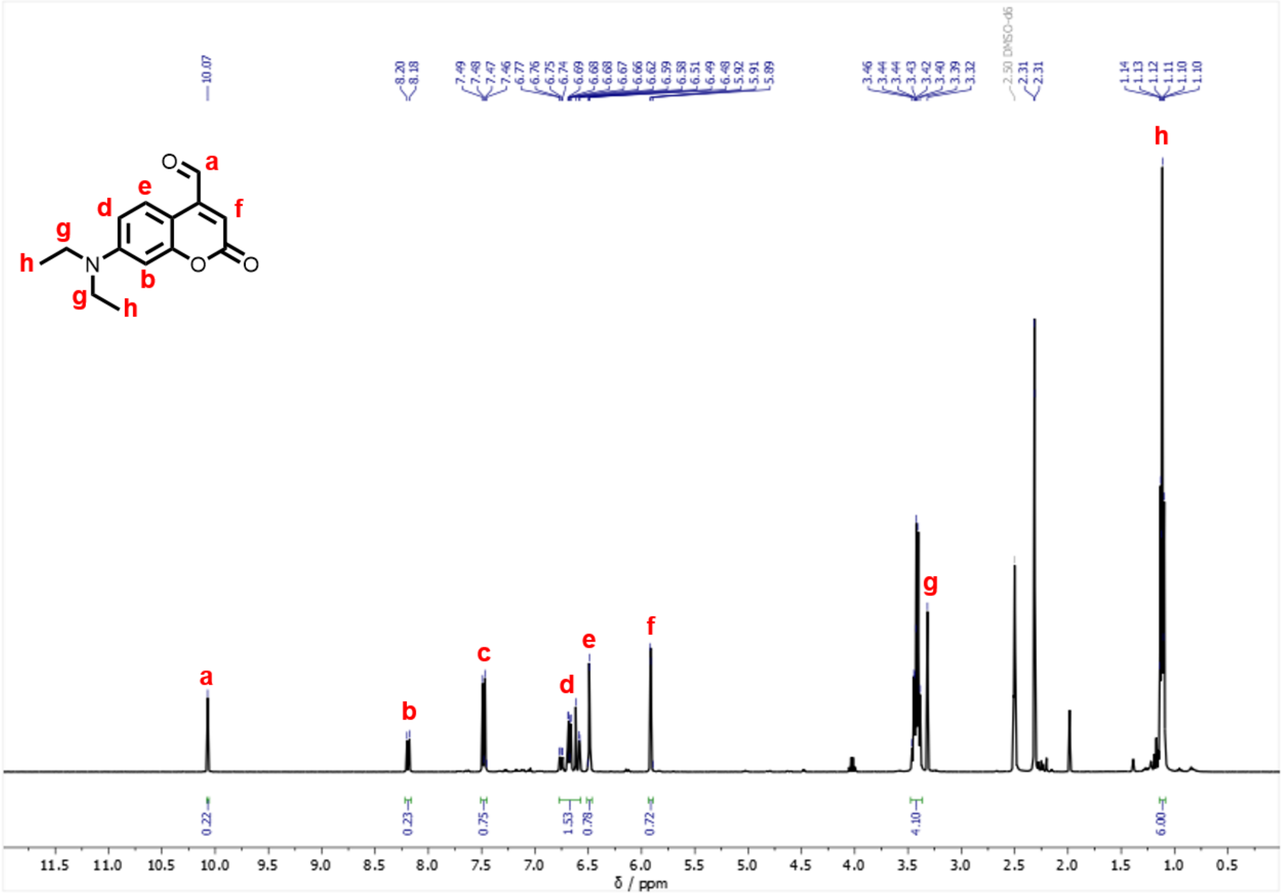

Figure S8: ^{1}H-NMR spectrum of compound **1c** (DMSO-d_6, 400 MHz).

The spectroscopic data is in agreement with literature.[1]

2.2 Synthesis of Compound 2

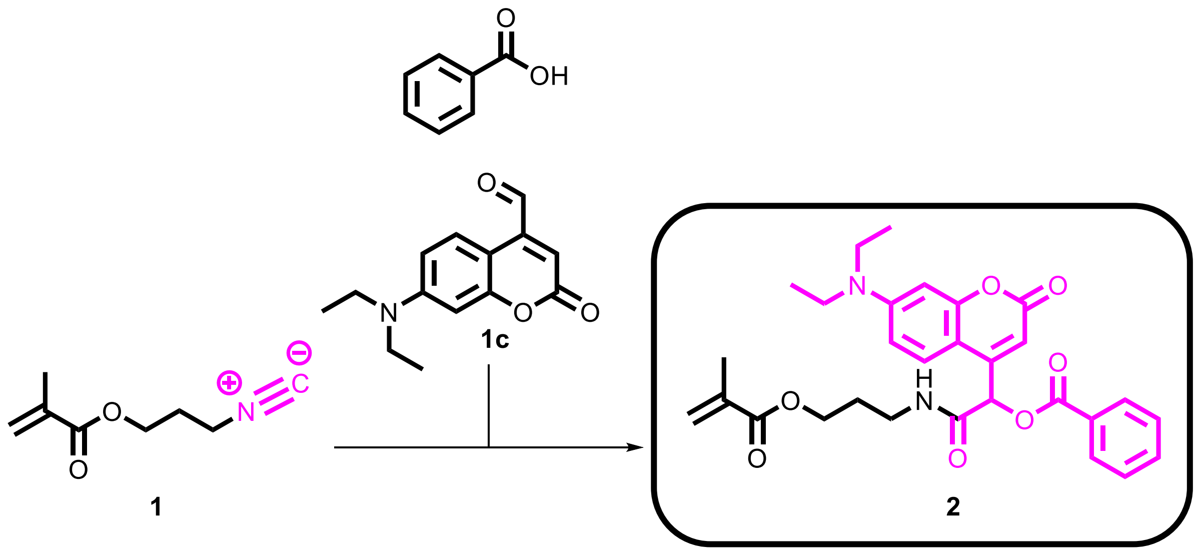

Scheme S2: Synthesis of Passerini coumarin derivative **2**: DCM, r. t., 16 h, 92%.

Compound 2

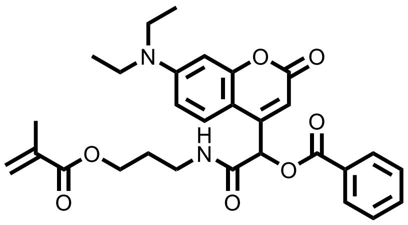

$C_{29}H_{32}N_2O_7$
520.5820 g mol^{-1}

130 mg **1** (848 µmol, 1.00 equiv.) was dissolved in 10 mL DCM. 245 mg **1c** (1.02 mmol, 1.20 equiv.), 125 mg benzoic acid (1.02 mmol, 1.20 equiv.) was added, and the reaction mixture was stirred for 16 h at ambient temperature. Subsequently, the solvent was removed under reduced pressure and the crude product purified via column chromatography (2% MeOH in DCM) to obtain 404 mg of the desired compound (777 µmol, 92%) as a yellow, fluffy solid.

$\mathbf{R}_f$ (2% MeOH in DCM) = 0.13.

^{1}H-NMR (600 MHz, DMSO-d_6): δ (ppm) = 8.55 (t, J = 5.8 Hz, 1H, NH), 8.07-8.05 (m, 2H, Ar-H), 7.81 (d, J = 9.0 Hz, 1H, Ar-H), 7.58 (t, J = 7.9 Hz, 2H, Ar-H), 7.50 (t, J = 7.8 Hz, 1H, Ar-H), 6.67 (dd, J = 9.2, 2.6 Hz, 1H, Ar-H), 6.55 (d, J = 2.5 Hz, 1H, Ar-H), 6.36 (s, 1H, CH), 6.24 (s, 1H, Ar-H), 5.99 (d, J = 1.9 Hz, 1H, CH), 5.63 (s, 1H, CH), 4.07-4.01 (m, 2H, CH_2), 3.43 (qd, J = 7.2, 2.4 Hz, 4H, 2x CH_2), 3.21 (q, J = 6.6. Hz, 2H, CH_2), 1.84 (d, J = 1.4 Hz, 3H, CH_3), 1.77 (p, J = 6.7 Hz, 2H, CH_2), 1.12 (t, J = 7.0 Hz, 6H, 2x CH_3).

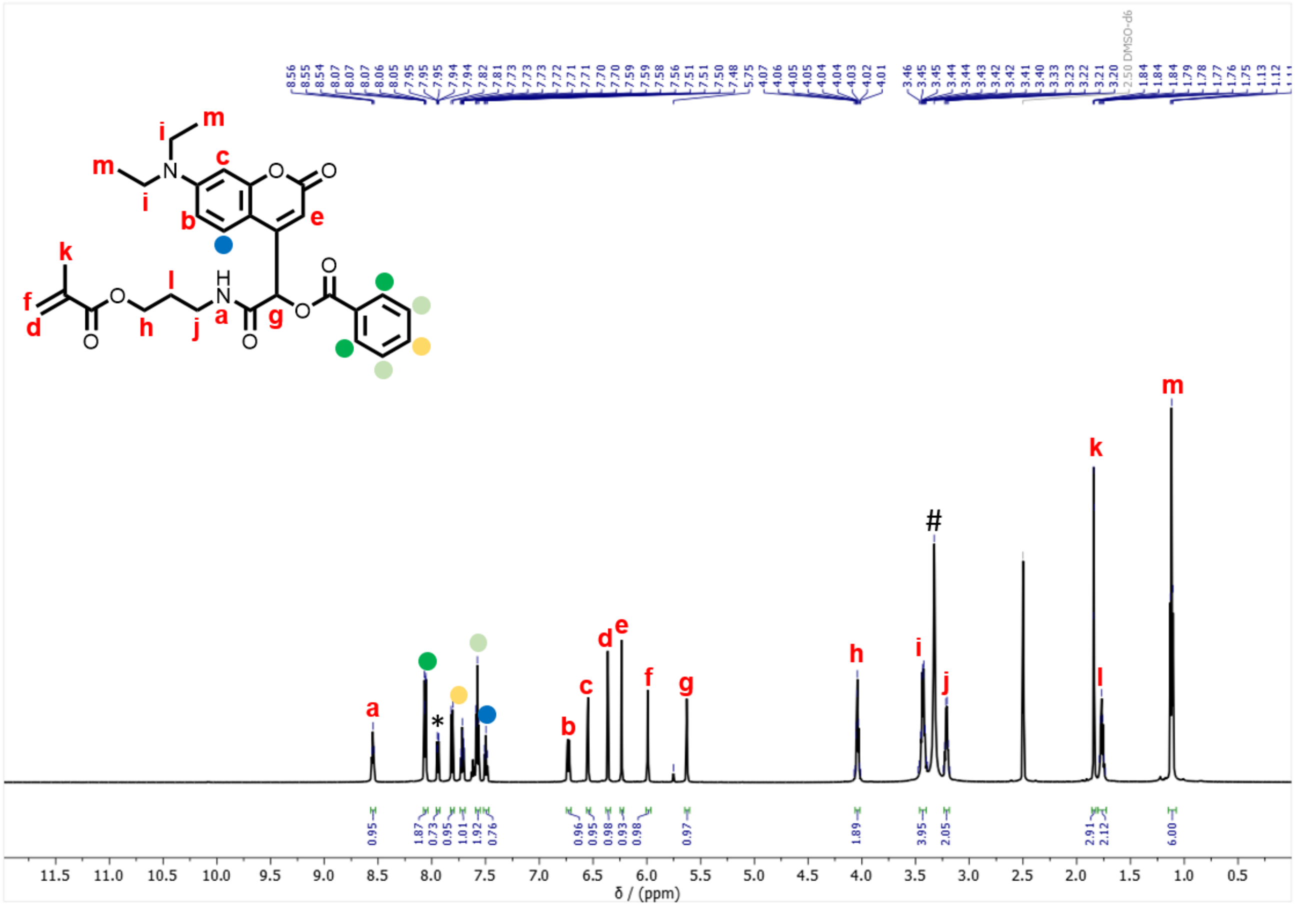

Figure S9: ^{1}H-NMR spectrum of compound **2** (DMSO-d_6, 600 MHz, *: DMF, #: water).

^{13}C-NMR (151 MHz, DMSO-d_6): δ (ppm) = 166.5 (s, 1C, *C*O), 165.7 (s, 1C, *C*O), 164.4 (s, 1C, *C*O), 160.6 (s, 1C, *C*O), 156.2 (s, 1C, *C*H), 150.5 (s, 1C, CH_{Ar}), 149.5 (s, 1C, CH_{Ar}), 135.8 (s, 1C, *C*H), 134.0 (s, 1C, CH_{Ar}), 129.3 (s, 2C, CH_{Ar}), 128.9 (s, 2C, CH_{Ar}), 128.5 (s, 1C, CH_{Ar}), 125.6 (s, 1C, CH_2), 108.8 (s, 1C, CH_{Ar}), 107.6 (s, 1C, CH_{Ar}), 105.3 (s, 1C, CH_{Ar}), 96.9 (s, 1C, CH_{Ar}), 72.8 (s, 1C, *C*H), 61.9 (s, 1C, CH_2), 44.0 (s, 2C, 2x CH_2), 35.8 (s, 1C, CH_2), 28.0 (s, 1C, CH_2), 18.0 (s, 1C, CH_3), 12.3 (s, 2C, 2x CH_3).

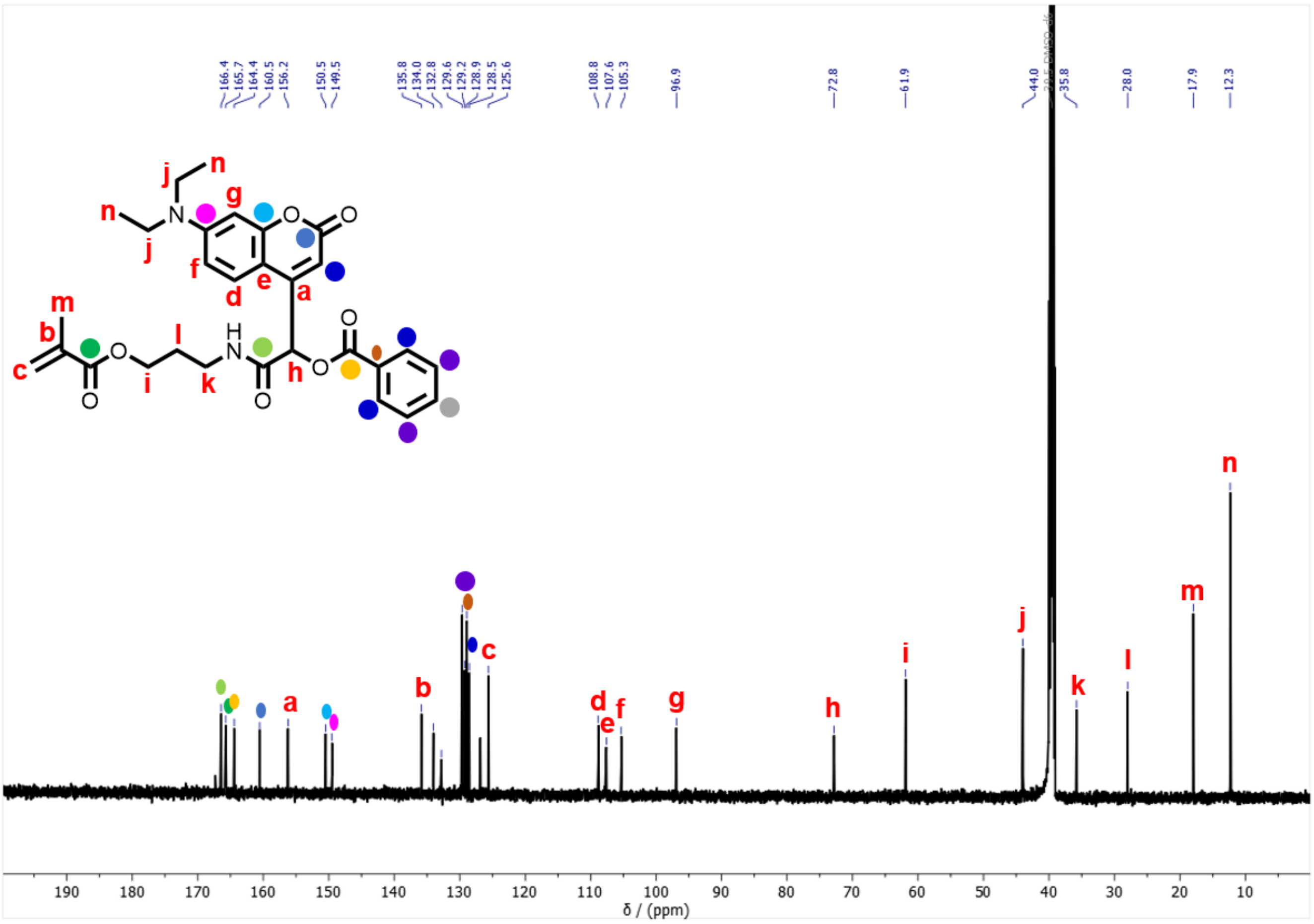

Figure S10: ^{13}C-NMR spectrum of compound **2** (DMSO-d_6, 151 MHz).

LC-MS:

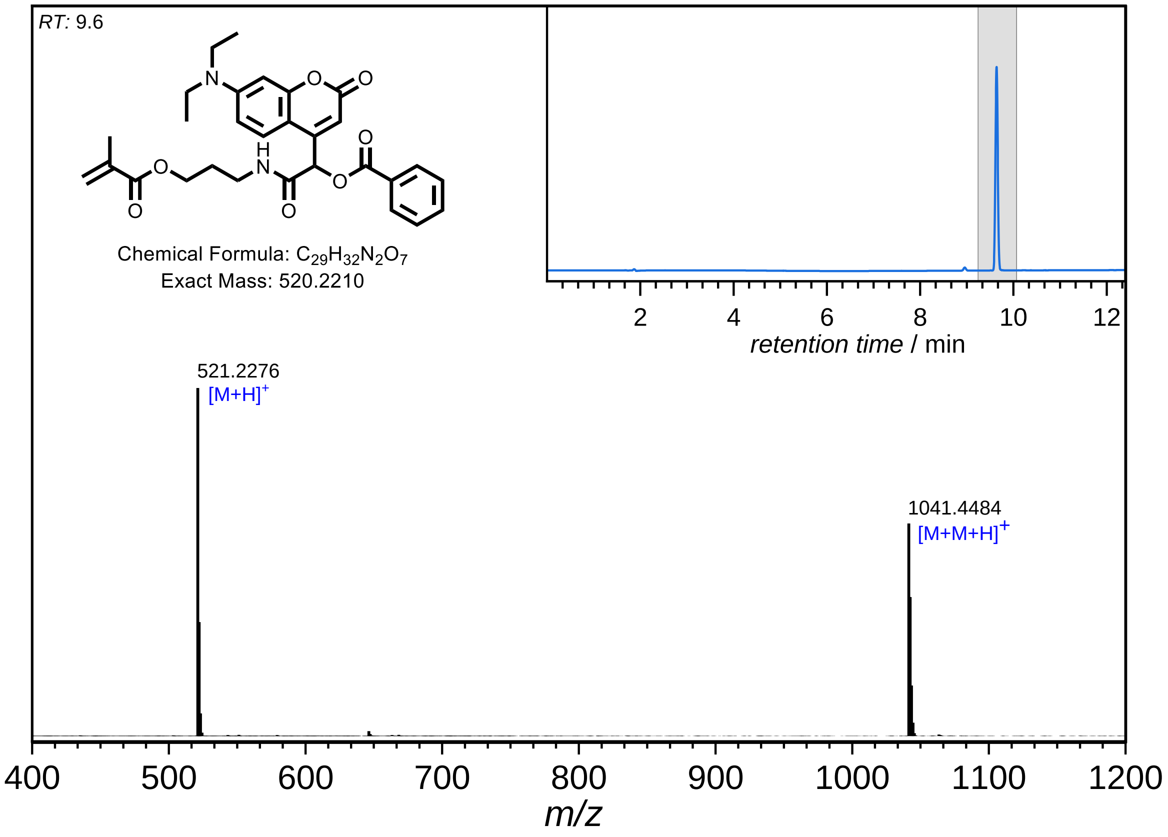

Figure S11: LC-MS analysis of compound **2**: m/z_{theo} = 520.2210 [$C_{29}H_{32}N_2O_7$], m/z_{exp} = 521.2276 $[M+H]^+$, 1041.4484 $[2M+H]^+$.

2.3 Synthesis of Compound 3

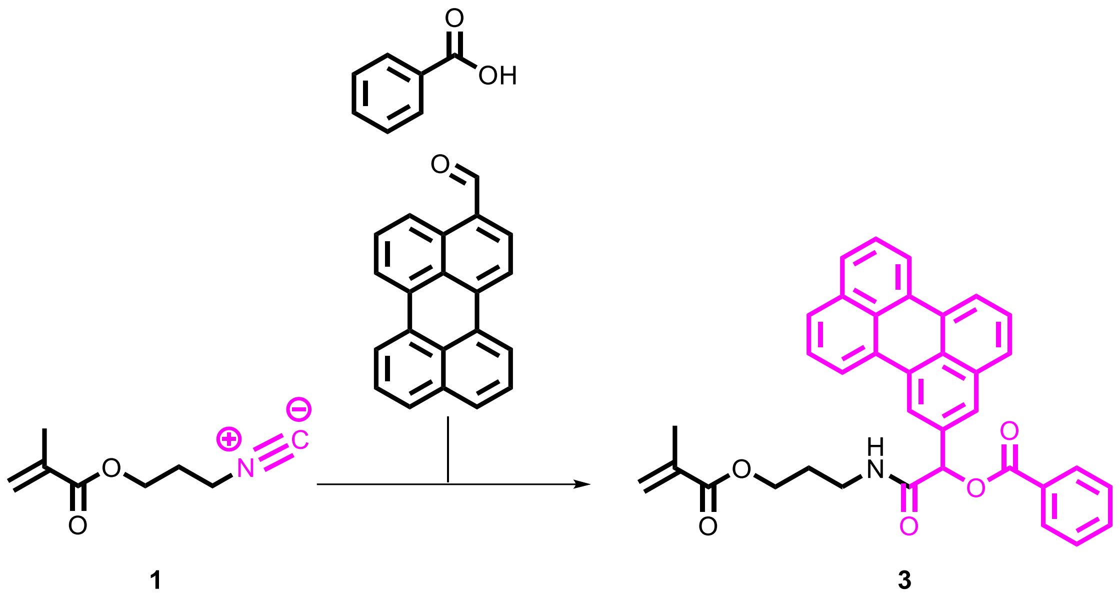

Scheme S3: Synthesis of Passerini perylene derivative **3**: $CHCl_3$, r. t., 16 h, 3%.

Compound 3

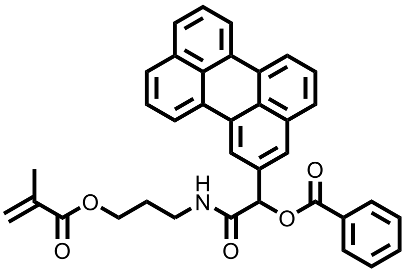

$C_{36}H_{29}NO_5$
555.6300 g mol^{-1}

196 mg perylenecarboxaldehyde (700 µmol, 1.20 equiv.) and 85.4 mg benzoic acid (700 µmol, 1.20 equiv.) was dissolved in 300 mL $CHCl_3$ in an amber flask. 90.0 mg **1** (583 µmol, 1.00 equiv.) was added and the reaction mixture stirred for 16 h at ambient temperature. The solvent was removed under reduced pressure and the crude product purified via two consecutive column chromatographic runs (first column: 1% MeOH in DCM, second column: 3% MeOH in DCM) to afford 9.00 mg of the desired product (16.2 mmol, 3%) as a fluffy, orange solid.

R*f* (first column: 1% MeOH in DCM) = 0.23.
R*f* (second column: 3% MeOH in DCM) = 0.75.

^{1}H-NMR (500 MHz, $CDCl_3$): δ (ppm) = 8.26 (d, J = 7.5 Hz, 1H, 1x Perylene-H), 8.23-8.20 (m, 3H, 3x Perylene-H), 8.17-8.14 (m, 3H, 1x Perylene-H, 2x CH_{Ar}), 7.73-7.70 (m, 3H, 3x CH_{Ar}), 7.59 (td, J = 7.5, 4.8 Hz, 2H, 2x Perylene-H), 7.48 (dt, J = 21.2, 7.7 Hz, 4H, 4x Perylene-H), 6.94 (s, 1H, CH), 6.57 (t, J = 6.3 Hz, 1H, NH), 6.05 (s, 1H, CH), 5.52 (t, J = 1.8 Hz, 1H, CH), 4.22 (dt, J = 11.8, 6.0 Hz, 1H, $CH_{2\text{-e1}}$), 4.13 (dt, J = 11.6, 5.9 Hz, 1H, $CH_{2\text{-e2}}$), 3.47-3.34 (m, 2H, CH_2), 1.89 (s, 3H, CH_3), 0.83 (d, J = 6.6, 2H, CH_2).

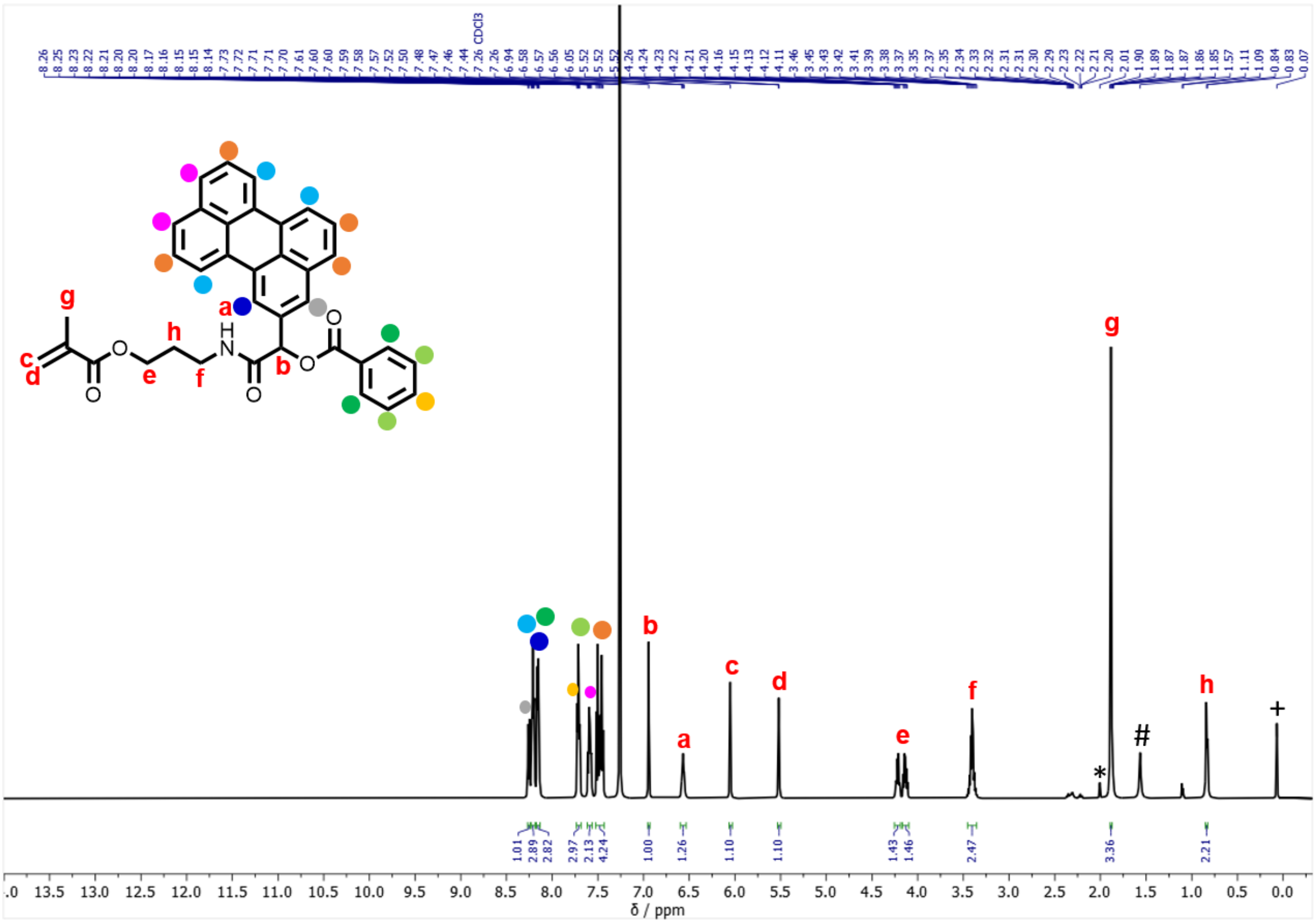

Figure S12: ^{1}H-NMR spectrum of compound **3** ($CDCl_3$, 500 MHz, *: acetone, #: water, +: TMS).

^{13}C-NMR (151 MHz, $CDCl_3$): *δ* (ppm) = 168.7 (s, 1C, *C*HO), 167.8 (s, 1C, *C*HO), 165.5 (s, 1C, *C*HO), 136.2 (s, 1C, *C*H), 134.7 (s, 1C, *C*H), 133.7 (s, 2C, Perylene-*C*H), 133.1 (s, 1C, Perylene-*C*), 132.9 (s, 1C, Perylene-*C*), 132.0 (s, 1C, Perylene-*C*), 131.0 (s, 1C, Benzoic Acid-*C*H), 130.2 (s, 2C, 2x Benzoic Acid-*C*H), 128.7 (s, 2C, 2x Benzoic Acid-*C*H), 128.7 (s, 4C, 3x Perylene-*C*, 1x *C*H_2), 128.2 (s, 1C, Perylene-*C*H), 126.8 (s, 1C, Perylene-*C*H), 123.8 (s, 1C, Perylene-*C*), 120.7 (s, 1C, Perylene-*C*), 119.8 (s, 1C, Perylene-*C*), 74.4 (s, 1C, *C*H), 61.9 (s, 1C, *C*H_2), 36.4 (s, 1C, *C*H_2), 28.9 (s, 1C, *C*H_2), 18.4 (s, 1C, *C*H_3).

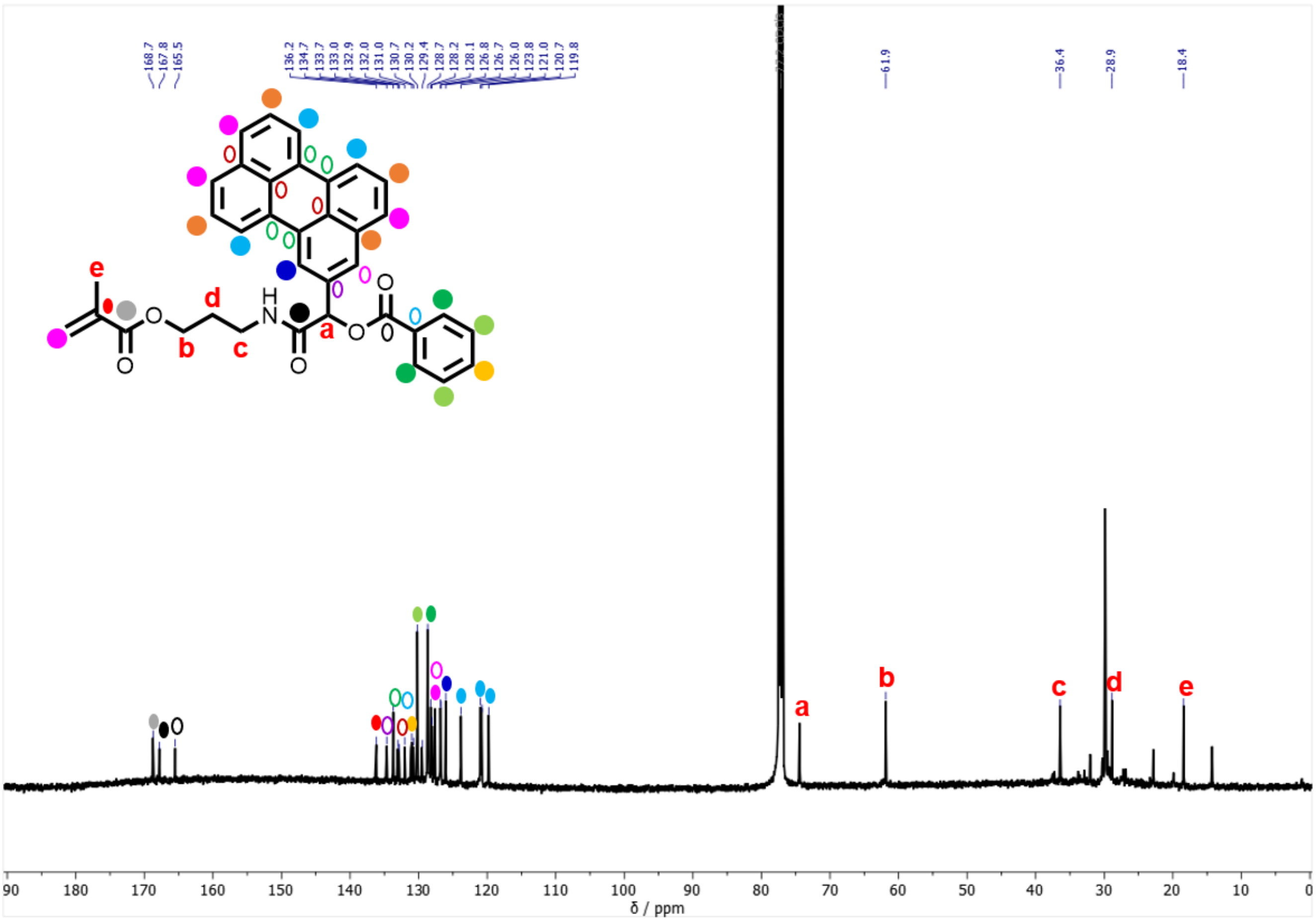

Figure S13: ^{13}C-NMR spectrum of compound **3** ($CDCl_3$, 151 MHz).

LC-MS:

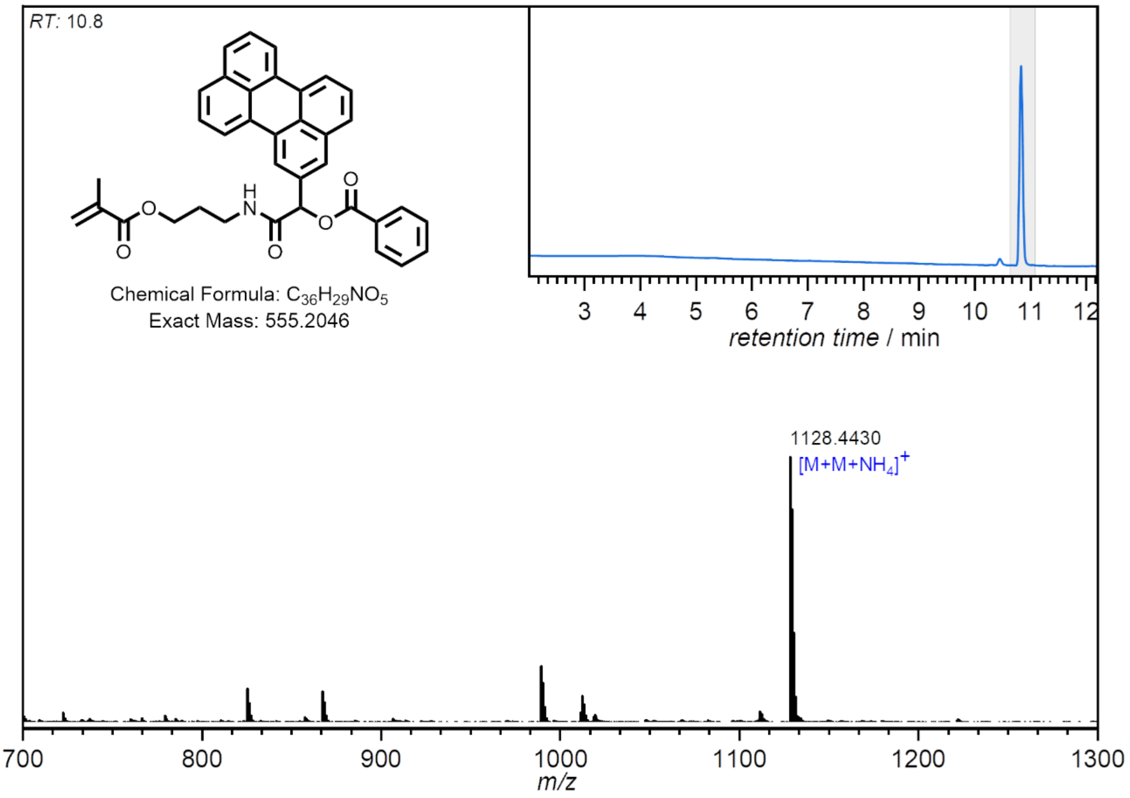

Figure S14: LC-MS analysis of compound **3**: m/z_{theo} = 555.2046 [$C_{35}H_{29}NO_5$], m/z_{exp} = 1128.4430 g mol^{-1} [$2M+NH_4$]$^+$.

3 Additional Data and Spectra

3.1 Compound 2

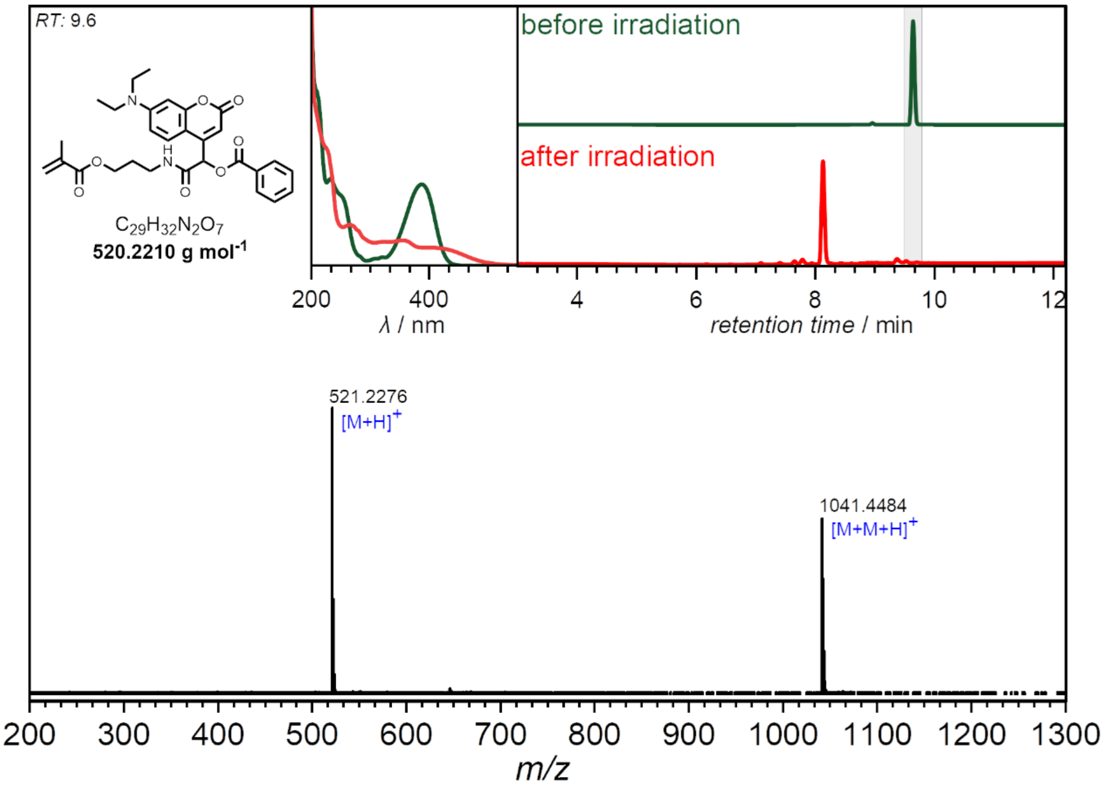

Figure S15: LC-MS analysis of compound **2** pre-irradiation: m/z_{theo} = 520.2210 [$C_{29}H_{32}N_2O_7$], m/z_{exp} = 521.2276 g mol^{-1} [M+H]$^+$ and 1041.4484 [2M+H]$^+$.

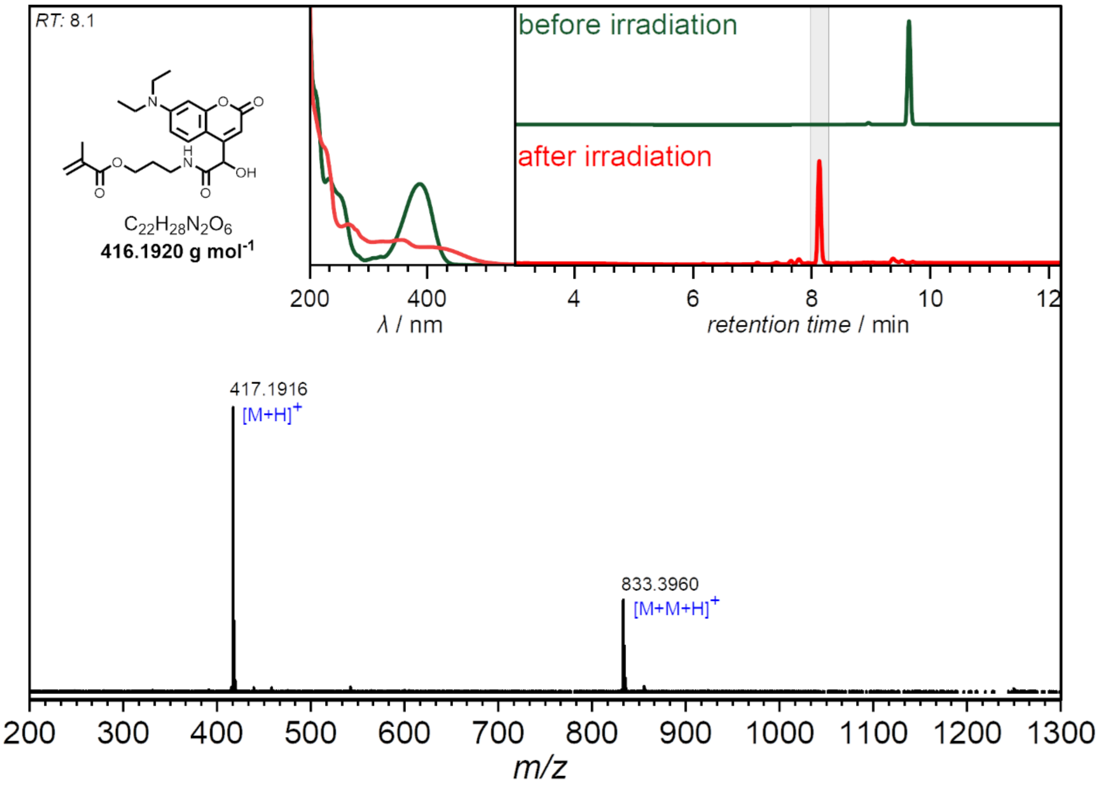

Figure S16: LC-MS analysis of compound **2** after irradiation for 2 h with a 400 nm LED, followed by purification: m/z_{theo} = 417.2026 for $[M+H]^+$ and 833.3979 for $[2M+H]^+$.

3.2 Compound 3

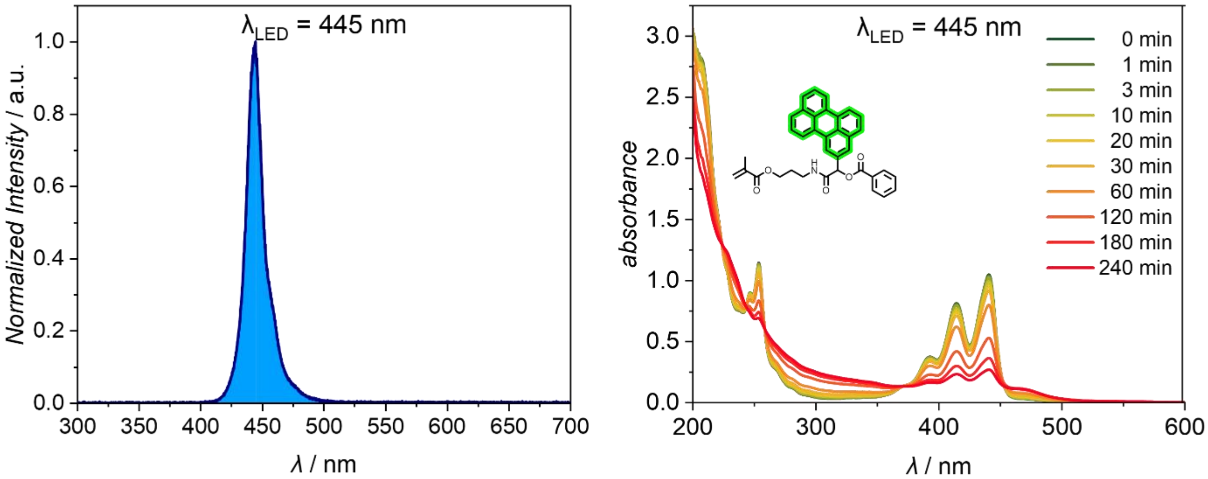

Figure S17: **left:** Normalized emission spectra of the 445 nm LED for the irradiation experiment of compound **3**. **Right:** UV/Vis absorbance changes of 40 µM perylene-monomer **3** over 4 h of irradiation at 20 °C with a 445 nm LED in MeCN. The absorbance spectra indicate the successful cleavage of the benzoic acid as during irradiation the absorbance maxima at λ_1 = 253 nm, λ_2 = 414 nm and λ_3 = 441 nm decrease and a new maximum at λ = 469 nm develops.

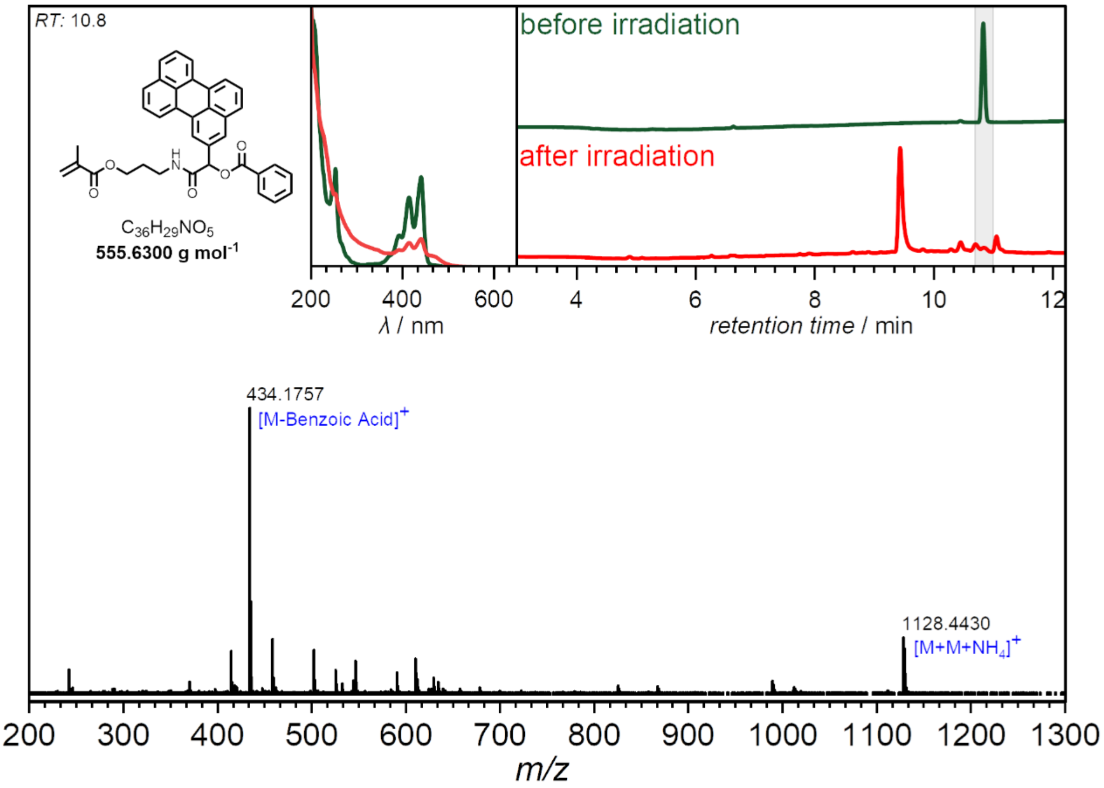

Figure S18: LC-MS analysis of compound **3** pre-irradiation: m/z_{theo}= 555.6300 [$C_{36}H_{29}NO_5$], m/z_{exp} = 1128.4430 [$2M+NH_4$]$^+$.

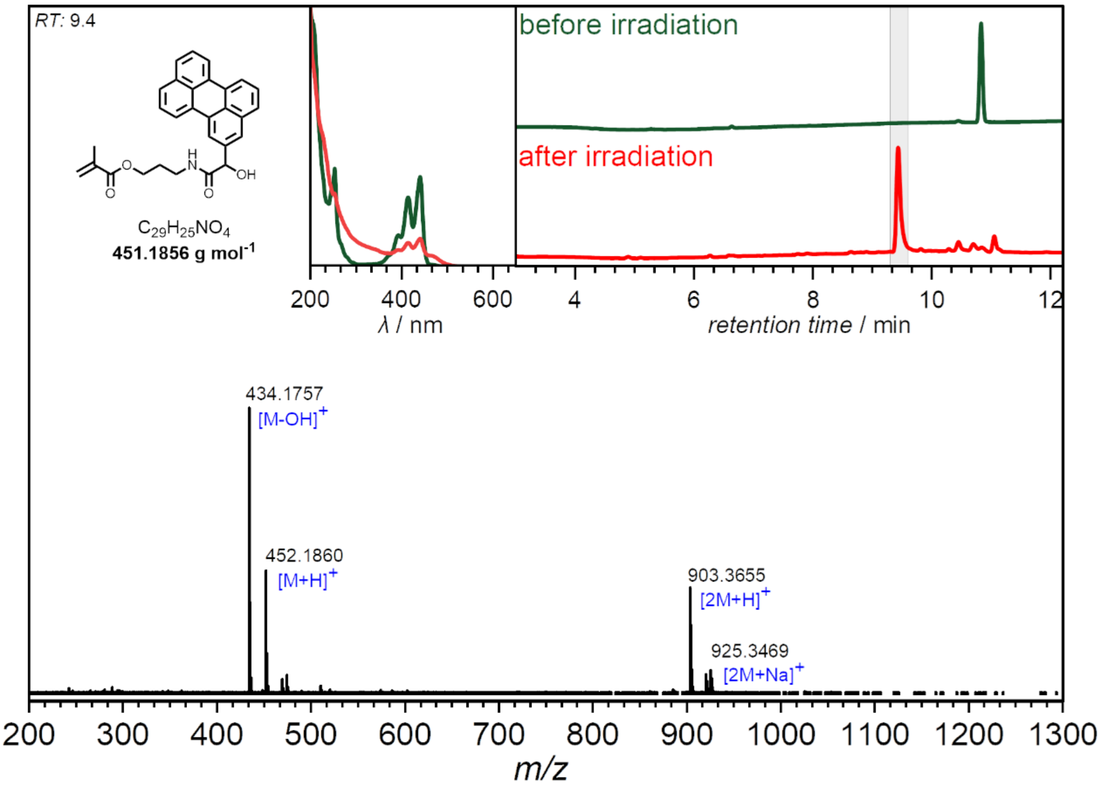

Figure S19: LC-MS analysis of compound **3** after irradiation for 4 h with a 445 nm LED, followed by purification: m/z_{theo} = 434.1751 for $[M\text{-}OH]^+$, m/z_{theo} = 452.1856 for $[M+H]^+$, m/z_{theo} = 903.3640 for $[2M+H]^+$, and m/z_{theo} = 925.3459 for $[2M+Na]^+$.

4 Tuneable Laser Experiments

Tunable laser experiments with a constant photon count at varied wavelengths were carried out according to a procedure published by our group.[2],3

4.1 Laser Set Up

All laser experiments were either conducted using a Coherent *Opolette* 355 OPO tunable laser system (QUT) or an *Innolas* SpitLight 600 OPO tunable laser system (KIT), operated at wavelengths between 300 nm and 650 nm with a full width half maximum of 7 ns and a repetition rate of 20 Hz. The emitted pulse, which has a flat-top spatial profile, was expanded to 6 mm diameter using focusing lenses and directed upwards using a prism. The spectral linewidth (FWHM) of the beam is 4-6 cm^{-1}. The energy of the laser pulses was downregulated by an attenuator (polarizer). The beam was redirected into the vertical cylindrical hole of a custom-made sample holder, which contains the sample during the experiments. For the irradiation experiments, all samples (300 µL degassed solution in MeCN) were prepared in 0.7 mL crimped glass vials by Supelco, Merck (Product Number: 24738-U). Since these glass vials were discontinued at Merck, the supplier was changed to Thermo Fisher (Clear Glass Vial, 7 x 40 mm, Flat Bottom, Product Number: C4008-741). The energy of the incident laser pulses was measured by an Energy Max OC power meter (Coherent) directly above the sample holder (immediately before each sample was inserted into the samples holder). Prism and samples holder are positioned such that the complete diameter of the hole of the sample holder was covered by the incident laser beam (**Figure S20**).

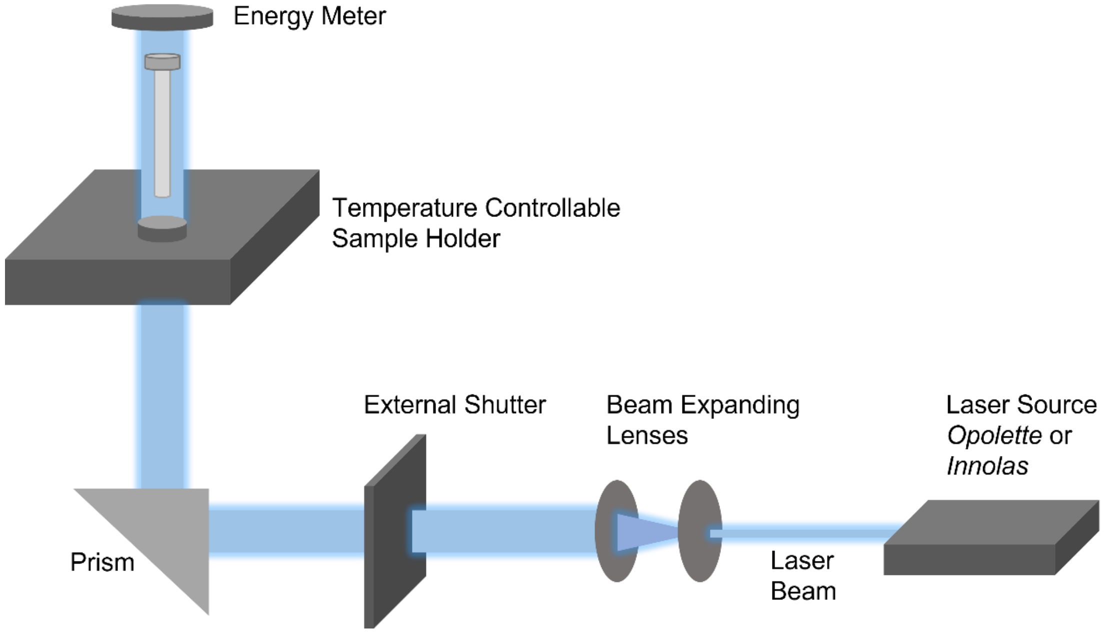

Figure S20: Experimental set-up for photochemical action plot measurements. Light from a monochromatic light source is expanded to cover the entire sample volume, passed through an external, mechanical shutter and directed onto the sample from below using a prism. The sample is suspended in an opaque aluminium block, and the energy delivered to the sample is monitored using an energy meter.

4.2 Control over Incident Photon Number

The number of photons n_P ([n_P] = mol) that a monochromatic laser pulse contains was determined from the laser pulse energy using the Planck-Einstein relation:

$$n_P = \frac{E_{Pulse} \cdot f_{rep} \cdot \lambda \cdot t}{h \cdot c \cdot [T_\lambda / 100]}$$

Where E_{Pulse} is the measured pulse energy above the aluminum block, λ is the wavelength of the incident radiation, f_{rep} is the laser repetition rate, t is the irradiation time, h is Planck's constant, c is the speed of light and T_λ is the wavelength dependent glass transmittance presented in **Figure S21.**

The wavelength dependent transmittance of the glass vials was determined experimentally using the above setup. Three glass vials were randomly selected as calibration vials. For varying wavelengths and in each case at a constant power output of the laser, the energy was measured both with and without the calibration vials fitted into the sample holder. The measured energy per pulse without a calibration vial in the sample holder is denoted as E_0 and the measured energy per pulse with a calibration vial in the sample holder as E_n. The transmittance was calculated as the ratio of E_n to E_0. The average transmittance over the measurements of the three vials (T_λ) was plotted together with the respective error (**Figure S21**).

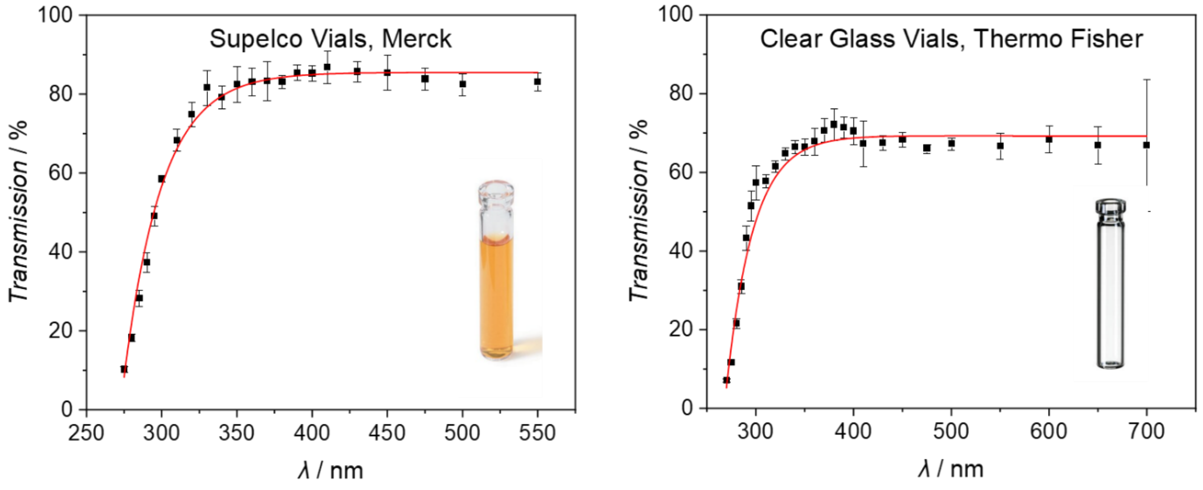

Figure S21: Left: Calibration of the glass vial transmittance of the Supelco Vials, purchased at Merck including a fit to obtain values that were not determined experimentally. **Right:** Calibration of the glass vial transmittance of the Clear Glass Vials, commercially available at Thermo Fisher including a fit to obtain values that were not determined experimentally.

4.3 Kinetic Measurements and Action Plot of Compound 2

*4.3.1 Results of the Kinetic Experiments of Compound **2***

The determination of the irradiation conditions was performed as described previously by our group.[3] Prior to the recording of an action plot, a kinetic analysis of the DEA-Cou-PMA **2** at the absorbance maximum (λ_{max} = 388 nm) of the chromophore was performed. Specifically, the coumarin derivative **2** was dissolved in MeCN at 80 µg mL^{-1} (147 µM). Sample solutions were freshly prepared every day and not kept overnight. The stock solution was prepared and bubbled with N_2 gas for 15 min, then 300 µL were withdrawn and added to capped laser vials (Supelco Vials, Merck) that had previously been flushed with N_2 for 5 min. Before starting the action plot measurements, the required number of photons to achieve 20-30% consumption of the starting material was determined. Therefore, 8 identical samples were irradiated for various time intervals (**Table S1**) with an identical photon flux, using the above described Coherent *Opolette* tuneable laser system. To determine the conversion of the starting material into the photodegradation product, UV/Vis spectroscopy was used. After irradiation, each sample was diluted with 800 µL MeCN followed by recording of the absorbance spectra.

First, the molar extinction coefficients of both the starting material (**Figure S22**, left) and the photodegradation product (**Figure S22**, right) – isolated by preparative thin layer chromatography – were determined by obtaining the average of a dilution series (**Figure S23**) using the Beer Lambert's law.

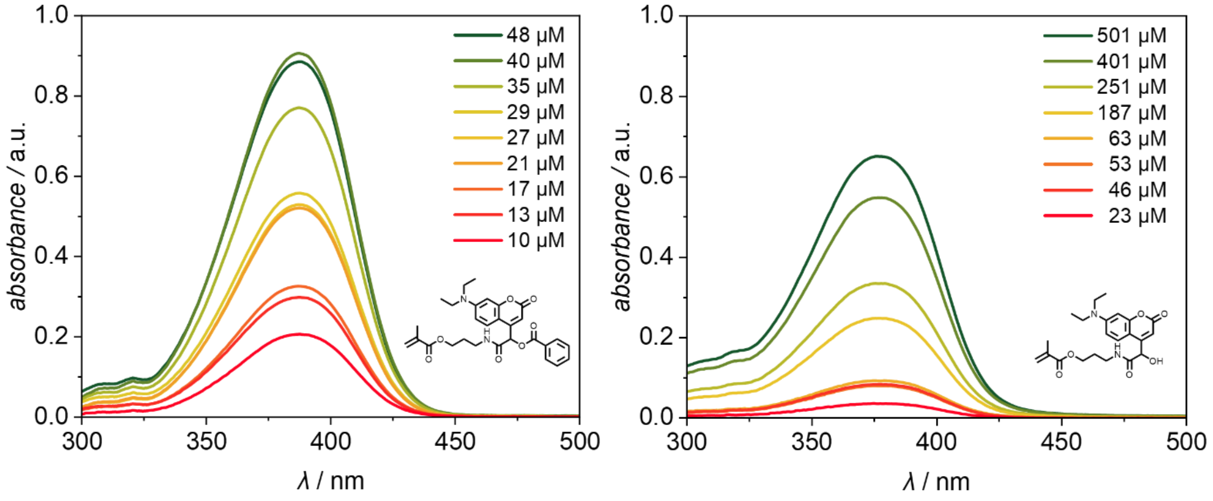

Figure S22: Left: Extinction coefficient spectra of the starting material coumarin-monomer **2. Right:** Extinction coefficient spectra of the photodegradation product.

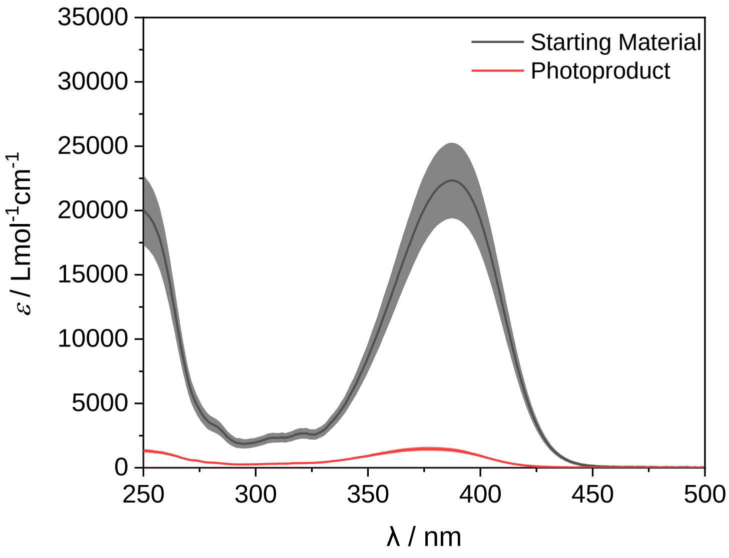

Figure S23: Molar extinction coefficient spectra for both the starting material and the photoproduct. Solid lines show the average calculated from a serial dilution, with the shaded area showing the error.

Given that the measured absorbance, *A*, spectrum at a given time, *t*, in the reaction of the starting material, SM, to the photoproduct, PP, can be deconvoluted in the equation:

$$A(t) = A_{\mathrm{SM}}(t) + A_{\mathrm{PP}}(t)$$

and that the absorbance, *A*, is given by:

$$A = \varepsilon c l$$

where ε is molar extinction coefficient, *c* is the concentration of the solution, and *l* is the path length of the solution, which is 1 cm in all of our experiments.

We are able to derive the equation:

$$A(t) = c_{\mathrm{SM}}\varepsilon_{\mathrm{SM}} + c_{\mathrm{PP}}\varepsilon_{\mathrm{PP}}$$

Thus, the concentration of each of the species present at time, *t*, is able to be calculated by fitting the measured spectrum to a linear combination of the two measured extinction coefficients. Thus, we selected the wavelength region 300 – 500 nm due to the significant blue shift observed upon formation of the photoproduct in this region, as well as the large difference in molar extinction coefficient between SM and PP – these factors improve the reliability of the fit.

We developed a solver tool using Microsoft Excel, of which a screenshot is shown in **Figure S24**, taking the molar extinction coefficient at each wavelength for the SM and PP (column B and C respectively), and combining them using cells C2 and C3 as scaling variables to afford the fit (column F). The error between the fit and the measured absorbance is subsequently assessed for each wavelength by taking the absolute value of the difference between the measured absorbance and the fit (column G), which is then summated in cell F2.

	A	B	C	D	E	F	G
1	**Molar extinction deconvoluter**						
2		cSM	0.00E+00		sum of error	6.394571901	
3		cPP	8.31E-05				
4							
5	Wavelength	e(Starting Material)	e(Photoproduct)		Measured absorbance	Fit	Error
6	500	5.273820944	1.562545125		0.0172	0.00012984	0.01707016
7	499	8.24715235	1.128599997		0.0176	9.37812E-05	0.017506219
8	498	4.911735284	1.167354861		0.0182	9.70016E-05	0.018102998
9	497	-3.864981857	1.959267839		0.0191	0.000162806	0.018937194
10	496	11.87571394	3.571444702		0.02	0.00029677	0.01970323
11	495	0.198929361	2.263721325		0.0207	0.000188104	0.020511896
12	494	-6.230303776	0.786181278		0.0212	6.53279E-05	0.021134672
13	493	3.72996438	1.223259084		0.0216	0.000101647	0.021498353
14	492	16.27483521	2.280784627		0.022	0.000189522	0.021810478
15	491	10.31708526	2.763045351		0.0227	0.000229596	0.022470404

Figure S24: Screenshot from the excel sheet used to deconvolute the absorption spectra after irradiation.

The data is fitted using a Generalized Reduced Gradient (GRG) non-linear algorithm with a convergence value of 0.0001 using the excel solver addon. The cell F2 is set to be minimised by changing the values in C2 and C3 (concentration of SM and PP), which were each set at $10 \cdot 10^{-6}$ mol dm^{-3} as a starting point. Once optimised, the concentrations can be extracted, and the conversion of starting material into photoproduct is given by the following equation,

$$conversion\ /\ \% = 100 - \frac{100 \cdot c_{\mathrm{SM}}}{c_{\mathrm{SM}} + c_{\mathrm{SM}}}$$

which is then directly plotted in the following reaction kinetics (and later action plot).

Table S1: Detailed specifications of the laser and results of the kinetic experiments for DEA-Cou-PMA **2**.

Sample	***λ* / nm**	***Irradiation time* / s**	***Pulse Energy* / µJ**	***# Photons***	***# Photons* / µmol**	***Consumption of Starting Material* / %**
1	388	0	0	0	0	0
2	388	30	323	3.49×10^{17}	0.579	22.5
3	388	60	320	6.97×10^{17}	1.16	46.6
4	388	90	326	1.05×10^{18}	1.74	58.8
5	388	120	295	1.39×10^{18}	2.31	70.6
6	388	180	285	2.09×10^{18}	3.48	80.6
7	388	240	282	2.79×10^{18}	4.58	85.3
8	388	300	272	3.49×10^{18}	5.80	88.9

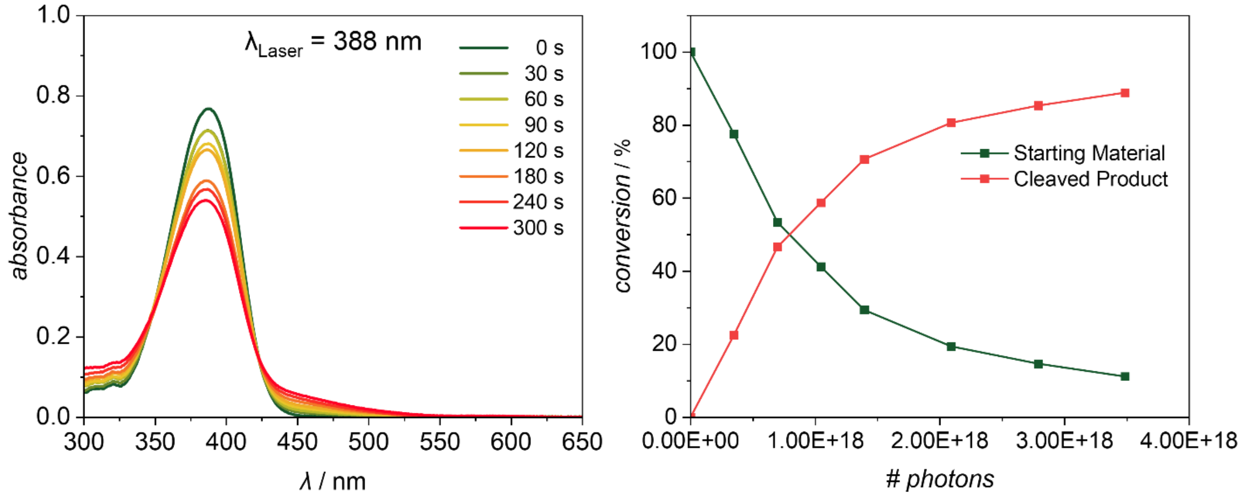

Figure S25: Left: UV/Vis absorbance spectra of samples 1-8 after irradiation with monochromatic laser light at λ_{Laser} = 388 nm to determine the reaction kinetics of DEA-Cou-PMA **2**. **Right:** Reaction kinetics of DEA-Cou-PMA **2**. To obtain approximately 25% consumption of the starting material, 0.66 µmol photons need to be deposited into the reaction system.

To obtain approximately 20-30% consumption of the starting material, 0.66 µmol photons (4.00 x 10^{17} photons) need to be deposited into the reaction system.

*4.3.2 Action Plot of Compound **2***

The kinetic experiments in chapter 4.3.1 revealed that for each indicated wavelength, 0.66 µmol of photons need to be deposited in the reaction system. For action plot measurements, compound **2** was dissolved in MeCN at 80 µg mL^{-1} (147 µM). Sample solutions were freshly prepared every day and not kept overnight. The stock solution was prepared and bubbled with N_2 for 15 minutes, then 300 µL were withdrawn and added to capped laser vials (Supelco Vials, Merck) that had previously been flushed with N_2 for 5 minutes. For each indicated wavelength, three samples à 300 µL were irradiated with the above describe Coherent *Opolette* tuneable laser system, followed by dilution in 800 µL MeCN and analysis via UV/Vis spectroscopy to calculate the consumption of the starting material (**Table S2**) via the previous introduced solver function in excel.

Table S2: Detailed specifications of the laser parameters and the calculations for the action plot of DEA-Cou-PMA **2**.

Sample	***λ / nm***	***Pulse Energy / µJ***	***Irradiation time / s***	***Consumption of Starting Material / %***	***Standard Error / %***
1	310	148	130	9.79	
2	310	147	132	9.86	
3	310	148	131	9.54	0.446
4	325	212	77	15.2	
5	325	210	78	15.7	
6	325	208	78	15.9	0.540
7	340	220	67	23.1	
8	340	219	67	23.9	
9	340	228	64	23.6	0.773
10	355	176	77	25.6	
11	355	183	75	25.6	
12	355	179	76	25.7	0.0521
13	370	227	63	24.8	
14	370	64	64	25.0	
15	370	64	64	24.0	0.694

Sample	*λ* / nm	*Pulse Energy* / μJ	*Irradiation time* / s	*Consumption of Starting Material* / %	*Standard Error* / %
16	385	194	76	21.0	16
17	385	191	80	21.1	
18	385	191	80	20.9	0.908
19	400	154	56	29.9	
20	400	146	58	29.5	
21	400	146	57	29.2	0.589
22	415	365	31	22.7	
23	415	343	33	22.8	
24	415	341	33	22.9	0.335
25	430	363	30	6.00	
26	430	334	32	6.25	
27	430	336	32	5.99	0.496
28	445	380	28	0	-
29	445	384	28	0	-
30	445	377	28	0	-
31	460	434	23	0	-
32	460	430	23	0	-
33	460	427	24	0	-
34	475	368	27	0	-
35	475	355	28	0	-
36	475	354	28	0	-
37	490	423	22	0	-
38	490	412	23	0	-
39	490	405	23	0	-
40	505	410	22	0	-
41	505	422	22	0	-
42	505	427	22	0	-
43	520	415	22	0	-
44	520	417	21	0	-
45	520	420	21	0	-

Once the wavelength-dependent reactivity was determined, a graph was plotted by overlaying the reactivity with the molar extinction spectrum, resulting in the chromophore's action plot (**Figure 2A** Main Article, **Figure S26**).

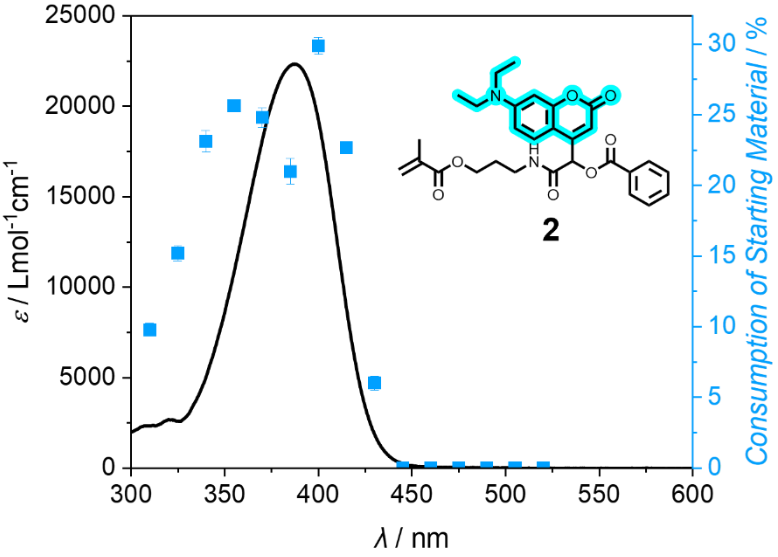

Figure S26: Molar extinction spectra of **2** overlayed with the action plot consumption of the starting material. For each indicated wavelength, 0.66 µmol photons were deposited into the reaction system (c_2 = 147 µM in MeCN).

4.4 Kinetic Measurements and Action Plot of Compound 3

4.4.1 *Results of the Kinetic Experiments of Compound **3***

Similar to compound **2**, a kinetic analysis of Per-PMA **3** at the absorbance maximum (λ_{max} = 441 nm) was performed prior to the recording of the action plot. Therefore, the perylene derivative **3** was dissolved in MeCN at 40 µg mL^{-1} (72 µM). Sample solutions were freshly prepared every day and not kept overnight. The stock solution was prepared and bubbled with N_2 for 15 min, then 300 µL were withdrawn and added to capped laser vials (Clear Glass Vials, Thermo Fisher) that had previously been flushed with N_2 for 5 min. Before starting action plot measurements, the required number of photons to achieve 20-30% consumption of the starting material should be determined. Therefore, 7 identical samples were irradiated for various time intervals (**Table S3**) with an identical photon flux, using the above described Coherent *Innolas* tuneable laser system. To determine the consumption of the starting material, LC-MS analysis was used. After irradiation of each sample for the respective time, they were filtered and 50 µL withdrawn into an LC-MS vial. Prior to analysis, 50 µL of benzene (*c* = 1 mg mL^{-1} in MeCN) was added as internal standard to determine the reaction kinetics (**Figure S27**).

Table S3: Detailed specifications of the laser parameters and results of the kinetic experiments for **Per-PMA 3**.

Sample	*λ* / nm	*Irradiation time* / s	*Pulse Energy* / µJ	*# Photons*	*# Photons* / µmol	*Consumption of Starting Material* / %
1	441	0	0	0	0	0
2	441	2	804	$4.27x\ 10^{18}$	7.09	25.0
3	441	5	744	9.88×10^{18}	16.4	45.2
4	441	10	689	1.83×10^{19}	30.4	58.4
5	441	20	630	3.35×10^{19}	50.6	77.0
6	441	30	542	$4.32x\ 10^{19}$	71.7	83.5
7	441	60	477	7.60×10^{19}	126	100

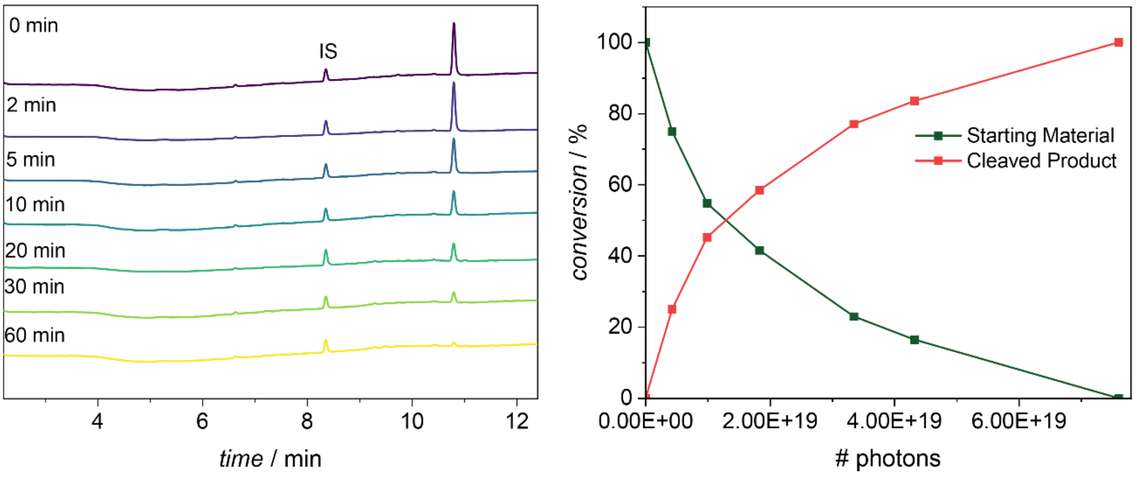

Figure S27: Left: LC traces of samples 1-7 after irradiation with monochromatic laser light at λ_{Laser} = 441 nm to determine the reaction kinetics of Per-PMA **3**. **Right:** Reaction kinetics of Per-PMA **3**. To obtain approximately 25% consumption of the starting material, 7.09 µmol photons need to be deposited into the reaction system.

To obtain approximately 20-30% consumption of the starting material, 7.09 µmol photons (4.27×10^{18} photons) need to be deposited into the reaction system.

4.4.2 Action Plot of Compound **3**

The kinetic experiments in chapter 4.4.1 revealed that for each indicated wavelength, 7.09 µmol of photons need to be deposited in the reaction system. For action plot measurements, compound **3** was dissolved in MeCN with the total concentration being 40 µg mL^{-1} (72 µM). Sample solutions were freshly prepared every day and not kept overnight. The stock solution was prepared and bubbled with N_2 for 15 min, then 300 µL were withdrawn and added to capped laser vials (Clear Glass Vials, Thermo Fisher) that had previously been flushed with N_2 for 5 min. For each indicated wavelength, three samples à 300 µL were irradiated with the above described Coherent *Innolas* tuneable laser system, followed by filtration. 50 µL of the irradiation sample were mixed with 50 µL benzene (c = 1 mg mL^{-1} in MeCN) and analyzed via LC-MS to calculate the consumption of the starting material via comparison of the ration of the peak integrals of the starting material and the internal standard (**Table S4**).

Table S4: Detailed specifications of the laser and the calculations for the action plot of Per-PMA **3**.

Sample	*λ* / nm	*Pulse Energy* / µJ	*Irradiation time* / min	*Consumption of Starting Material* / %	*Standard Error* / %
1	310	853	04:02 s	20.5	
2	310	797	04:19 s	19.4	
3	310	815	04:13 s	21.6	1.096
4	325	870	03:20 s	17.4	
5	325	914	03:10 s	18.9	
6	325	898	03:13 s	19.6	1.259
7	340	846	03:04 s	29.8	
8	340	846	03:04 s	28.7	
9	340	800	03:15 s	30.7	0.839
10	355	1000	02:25 s	31.8	
11	355	1000	02:25 s	30.7	
12	355	984	02:27 s	32.1	0.904
13	370	858	02:39 s	35.5	
14	370	867	02:38 s	34.9	
15	370	883	02:35 s	36.3	0.572
16	385	600	03:37 s	32.7	
17	385	687	03:10 s	31.3	
18	385	717	03:02 s	34.2	1.173
19	400	550	03:47 s	34.9	
20	400	611	03:24 s	30.8	
21	400	592	03:31 s	39.9	3.767
22	415	1100	01:49 s	26.6	
23	415	1020	01:59 s	27.7	
24	415	1090	01:50 s	29.5	1.576
25	430	1080	01:47 s	24.7	
26	430	1120	01:43 s	23.9	
27	430	1120	01:43 s	25.5	0.802
28	445	1180	01:35 s	16.3	
29	445	1220	01:32 s	18.4	
30	445	1190	01:34 s	14.2	2.095
31	460	1010	01:47 s	10.02	
32	460	1070	01:41 s	9.37	
33	460	1140	01:35 s	11.2	0.698
34	475	1110	01:34 s	4.08	
35	475	1140	01:32 s	6.19	
36	475	1130	01:32 s	7.21	0.886
37	490	1140	01:29 s	3.33	

Sample	*λ* / nm	*Pulse Energy* / µJ	*Irradiation time* / min	*Consumption of Starting Material* / %	*Standard Error* / %
38	490	1130	01:30 s	4.09	38
39	490	1090	01:33 s	3.98	0.371
40	505	992	01:39 s	1.06	
41	505	995	01:39 s	1.89	
42	505	991	01:39 s	2.09	0.151
43	520	1010	01:34 s	1.94	
44	520	1050	01:31 s	3.02	
45	520	1070	01:29 s	1.70	1.83
46	535	1080	01:26 s	2.85	
47	535	1100	01:24 s	2.55	
48	535	1070	01:26 s	4.37	1.59
49	550	1070	01:24 s	2.55	
50	550	1080	01:23 s	2.22	
51	550	1070	01:24 s	2.96	0.331
52	565	1050	01:24 s	2.50	
53	565	1090	01:21 s	1.98	
54	565	1020	01:26 s	0.894	0.411
55	580	1020	01:24 s	1.37	
56	580	1040	01:23 s	0.756	
57	580	1020	01:24 s	2.66	1.02
58	595	996	01:28 s	1.13	
59	595	993	01:28 s	2.06	
60	595	996	01:28 s	0.896	0.924

Once the wavelength-dependent reactivity was determined, a graph was plotted overlaying the reactivity with the molar extinction spectrum, resulting in the chromophore's action plot (**Figure 2C** Main Article, **Figure S28**).

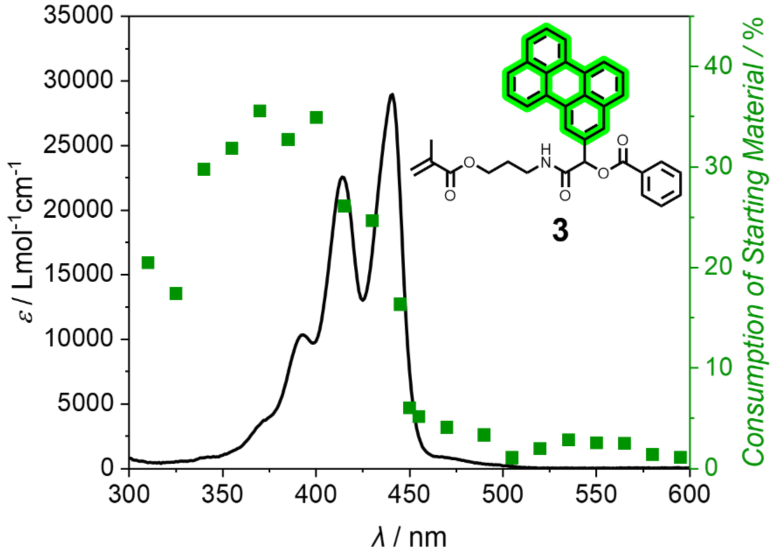

Figure S28: Molar extinction spectra of **3** overlayed with the action plot consumption of the starting material. For each indicated wavelength, 7.09 µmol photons were deposited into the reaction system (c_3 = 72 µM in MeCN).

5 Compound 4

5.1 Synthesis of Compound 4

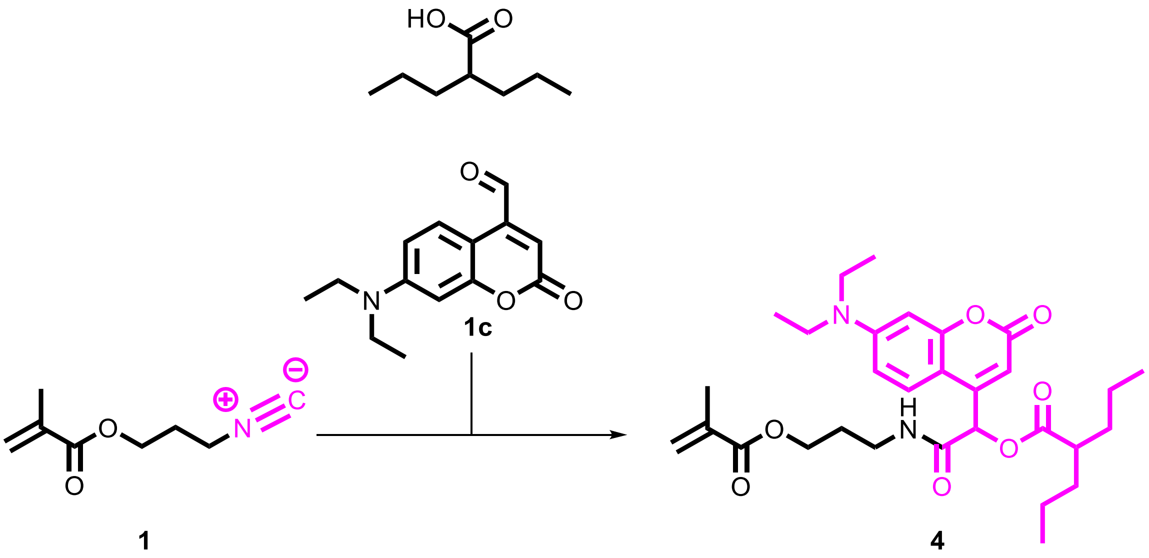

Scheme S4: Synthesis of Passerini coumarin derivative **4**: DCM, r. t., 16 h, 22%.

Compound 4

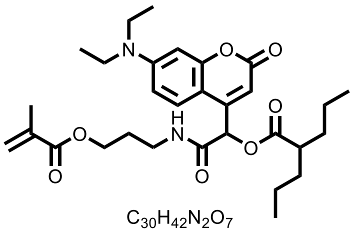

$C_{30}H_{42}N_2O_7$
542.6730 g mol^{-1}

90 µL valproic acid (81.4 mg, 564 µmol, 1.10 equiv.) and 153 mg **1c** (624 µmol, 1.20 equiv.) was dissolved in 20 mL DCM in an amber flask. 78.9 mg of **1** (515 µmol, 1.00 equiv.) was added and the reaction mixture stirred for 16 h at ambient temperature. Subsequently, the reaction mixture was washed with water (2 x 10 mL), saturated $NaHCO_3$ solution (3 x 10 mL) and water (3 x 10 mL), followed by removal of the solvent under reduced pressure. The crude product was purified twice via column chromatography (first column: 1% MeOH in DCM, second column: 2% MeOH in DCM) to obtain 61.5 mg of the desired compound (113 µmol, 22%) as a highly viscose, yellow oil.

R_f (first column: 1% MeOH in DCM) = 0.19 – The product was stained using $KMnO_4$- solution.
R_f (second column: 2% MeOH in DCM) = 0.18 – The product was stained using $KMnO_4$-solution.

^{1}H-NMR (400 MHz, DMSO-d_6): δ (ppm) = 8.41 (t, J = 5.7 Hz, 1H, NH), 7.70 (d, J = 9.2 Hz, 1H, Ar-H), 6.68 (dd, J = 9.2, 2.6 Hz, 1H, Ar-H), 6.53 (d, J = 5.53 Hz, 1H, Ar-H), 6.11 (d, J = 10.3 Hz, 2H, CH_2), 6.00 (dd, J = 1.9, 1.0 Hz, 1H, Ar-H), 5.64 (p, J = 1.7 Hz, 1H, Ar-H), 4.02 (t, J = 6.4 Hz, 2H, CH_2), 3.43 (q, J = 7.0 Hz, 4H, 2x CH_2), 3.18 (q, J = 6.5 Hz, 2H, CH_2), 2.54 (s, 1H), 1.85 (t, J = 1.3 Hz, 3H, CH_3), 1.74 (p, J = 6.6. Hz, 2H, CH_2), 1.62-1.49 (m, 2H, CH_2), 1.43 (dddd, J = 12.9, 10.8, 6.2, 3.3 Hz, 2H, CH_2), 1.34-1.21 (m, 4H, 2x CH_2), 1.11 (t, J = 7.0 Hz, 6H, 2x CH_3), 0.83 (dt, J = 15.8, 7.3 Hz, 6H, 2x CH_3).

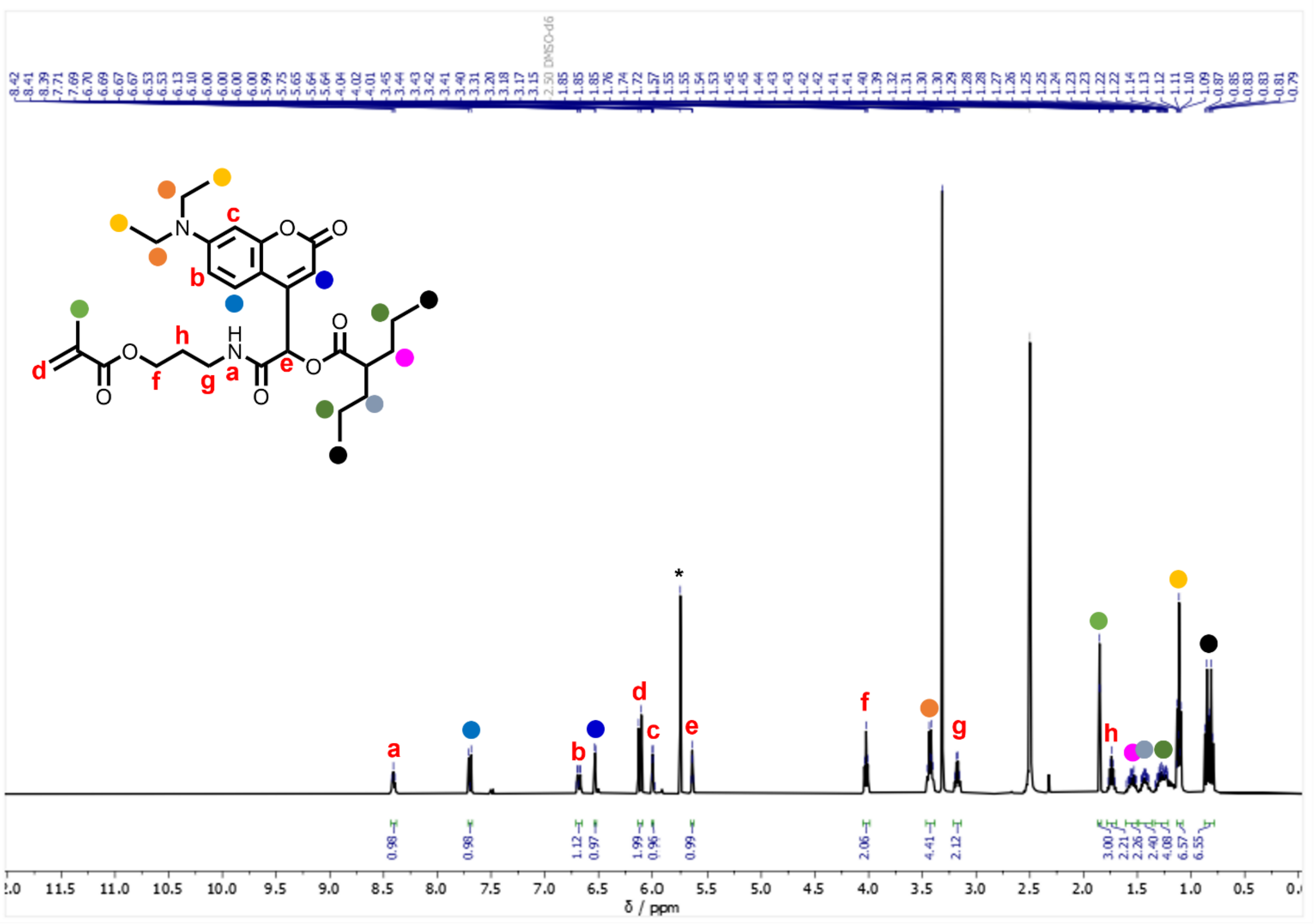

Figure S29: ^{1}H-NMR spectrum of compound **4** (DMSO-d_6, 400 MHz, *: DCM).

^{13}C-NMR (101 MHz, $CDCl_3$): δ (ppm) = 174.4 (s, 1C, *C*HO), 167.8 (s, 1C, *C*HO), 166.5 (s, 1C, *C*HO), 162.0 (s, 1C, *C*HO), 151.0 (s, 1C, *C*H), 150.2 (s, 1C, *C*H), 136.1 (s, 1C, *C*H), 126.8 (d, 1C, Coumarin-*C*H), 126.1 (s, 1C, Coumarin-*C*H), 109.0 (s, 1C, Coumarin-*C*), 106.7 (s, 1C, Coumarin-*C*H), 106.3 (s, 1C, Coumarin-*C*H), 97.7 (s, 1C, *C*H), 71.1 (s, 1C, *C*H_2), 61.6 (s, 1C, *C*H_2), 45.2 (s, 2C, 2x *C*H_2), 36.2 (s, 1C, *C*H), 34.4 (s, 2C, 2x C*H*$_2$), 28.8 (s, 1C, *C*H_2), 20.7 (s, 1C, *C*H_2), 20.6 (s, 1C, *C*H_3), 18.4 (s, 1C, *C*H_3), 14.0 (s, 2C, 2x *C*H_3), 12.6 (s, 2C, 2x *C*H_3).

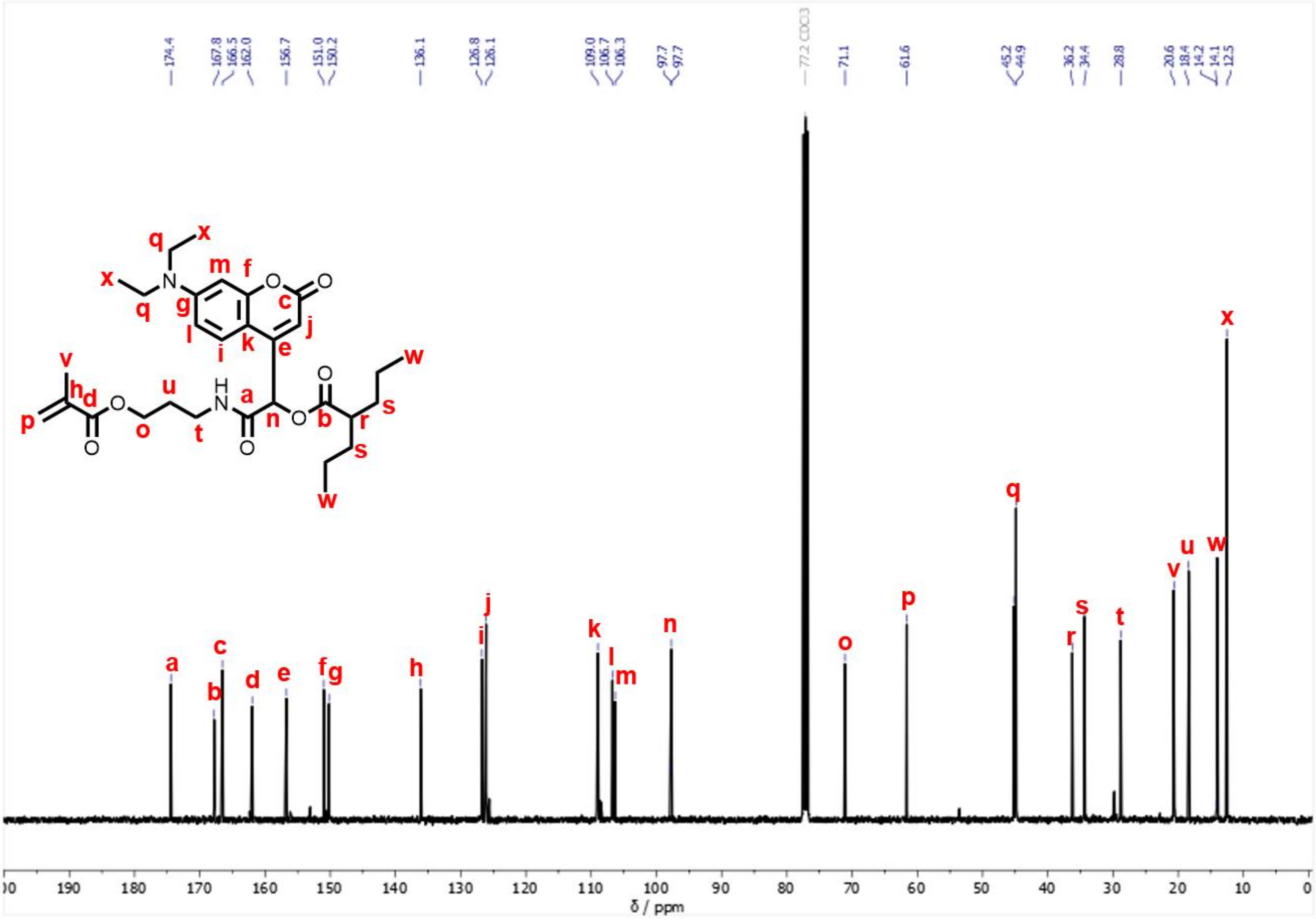

Figure S30: ^{13}C-NMR spectrum of compound **4** ($CDCl_3$, 101 MHz).

ESI-HRMS:

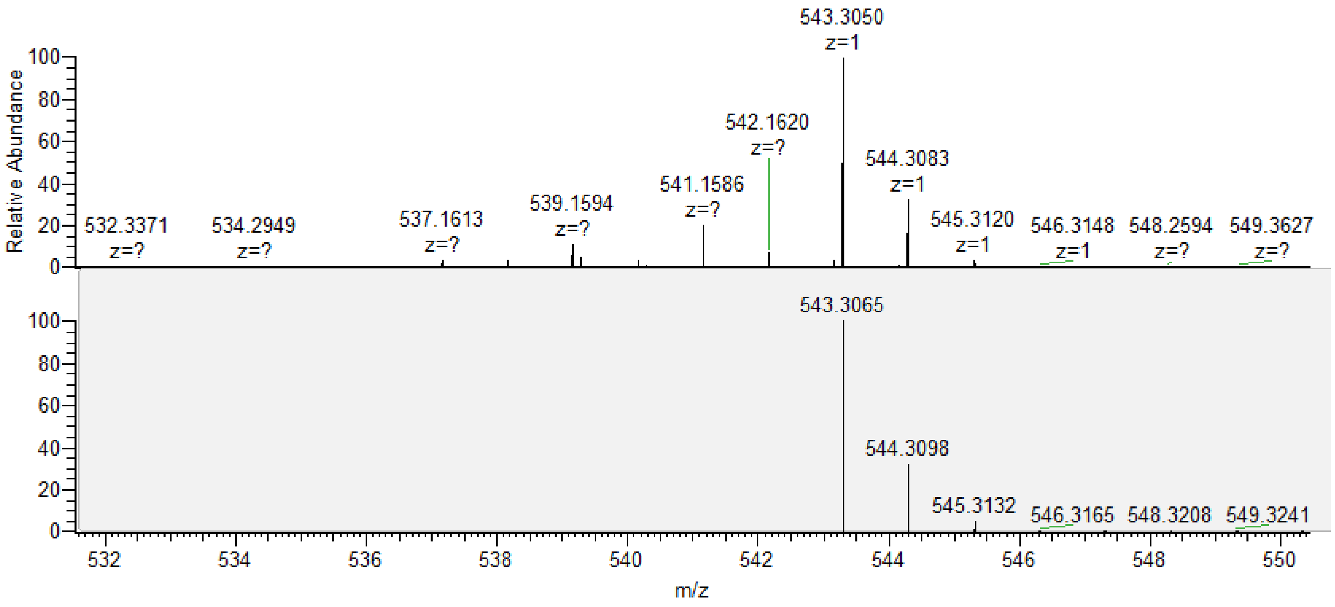

Figure S31: ESI-HRMS analysis of compound **4**: m/z_{theo} = 542.3065 [$C_{30}H_{42}N_2O_7$], m/z_{exp} = 543.3050 $[M+H]^+$.

5.2 Analysis of Compound 4

To obtain insight into the cleavage of the valproic acid during irradiation, initial experiments using a 385 nm LED were carried out. Despite potential deviations in the absorption maximum from the reactivity maximum, as indicated by research with action plots, these experiments provide a preliminary indication of how the compound reacts during irradiation.

Thus, a 150 µM solution of compound **4** was prepared and irradiated with a 385 nm LED at 20 °C for a total of ten minutes. UV/Vis measurements (**Figure S32**, left) and fluorescence emission spectra (**Figure S32**, right) were initially taken in 30 s and later in 60 s intervals.

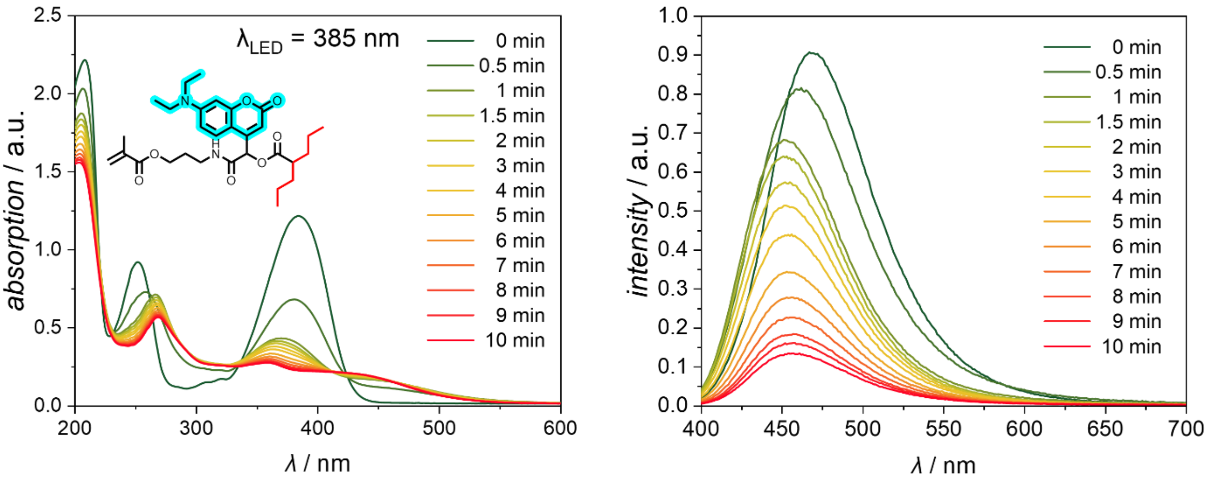

Figure S32: Left: UV/Vis absorbance changes of 150 µM compound **4** over 10 minutes of irradiation with a 385 nm LED in MeCN. **Right:** Fluorescence emission spectra of 150 µM compound **4** over 10 minutes of irradiation with a 385 nm LED.

Pre-irradiation (**Figure S32** left, green line), the compound shows three absorbance maxima at λ_1 = 208 nm, λ_2 = 250 nm and λ_3 = 384 nm. Over the course of irradiation (**Figure S32** left, red line), the absorbance maxima decrease and two isobestic points develop at λ_1 = 335 nm and λ_2 = 423 nm, indicating successful cleavage of the valproic acid. Furthermore, a decrease of absorption intensity occurs, due to substrate cleavage. Moreover, general bleaching of chromophores contributes to the decrease of intensity. The main signals are shifting, namely, the local maximum at λ = 385 nm undergoes a sligtht hypsochromic shift to λ = 357 nm, whereas the short-wavelength signal at 250 nm is shifted bathochromically to λ = 269 nm The blue-shift can be explained by a transition from the ground-state to the lowest $^1(\pi, \pi^*)$ excited singlet state of coumarin as a chromophore. The electron delocalization energy is higher before the irradiation due to the presence of the carboxyl group. By releasing the acid, the size of the conjugated system is reduced, resulting in the shift of the absorption peak. The bathochromic shift of the second peak at λ = 250 nm is attributable to another excitation process, in which the energy between orbitals increases due to the photorelease through irradiation.

Simultaneously with the absorption measurements, fluorescence emission spectra of compound **4** (**Figure S32** right) were recorded in the same irradiation time intervals as the UV/Vis absorption spectra. Pre-irradiation (**Figure S32** right, green line), the compound shows a fluorescent maximum at λ = 469 nm. Over the course of irradiation, the measured fluorescence intensity decreases due to the bleaching of the chromophore. Furthermore, a blue-shift of the fluorescent maximum at λ = 469 nm to λ = 451 nm can be detected, traced back to the same change in delocalization space in the free coumarin derivate, compared to the caged compound.

Both UV/Vis and fluorescence emissin spectra reveal that the cleavage reaction proceeds fast and is completed in an efficient manner during the first minute.

5.2.1 *Results of the Kinetic Experiments of Compound **4***

Similar to compounds **2** and **3**, a kinetic analysis of compound **4** at the absorbance maximum (λ_{max} = 384 nm) was performed prior to the recording of the action plot. Therefore, the coumarin derivative **4** was dissolved in MeCN with the total concentration being 80 µg mL^{-1} (150 µM). Sample solutions were freshly prepared every day and not kept overnight. The stock solution was prepared and bubbled with N_2 for 15 min, then 300 µL were withdrawn and added to capped laser vials (Supelco Vials, Merck) that had previously been flushed with N_2 for 5 min. Before starting action plot measurements, the required number of photons to achieve 20-30% consumption of the starting material should be determined to allow for a buffer in the conversion range. Therefore, 12 identical samples were irradiated for various time intervals (**Table S5**) with an identical photon flux, using the above described Coherent *Innolas* tuneable laser system. To determine the consumption of the starting material, LC-MS analysis was employed. After irradiation of each sample for the respective time, they were filtered and 50 µL withdrawn into an LC-MS vial. Prior to analysis, 50 µL of benzene (c = 1 mg mL^{-1} in MeCN) was added as internal standard to determine the reaction kinetics (**Figure S33**).

Table S5: Detailed specifications of the laser and results of the kinetic experiments for compound **4**.

Sample	*λ* / nm	*Irradiation time* / s	*# Photons*	*# Photons* / µmol	*Consumption of Starting Material* / %
1	384	0	0	0	0
2	384	20	4.89×10^{17}	0.812	13.24
3	384	30	7.40×10^{17}	1.23	16.91
4	384	40	1.01×10^{18}	1.67	23.61
5	384	50	1.30×10^{18}	2.16	26.87
6	384	60	1.55×10^{18}	2.57	40.90
7	384	90	2.27×10^{18}	3.77	54.92
8	384	120	2.97×10^{18}	4.93	66.05
9	384	150	3.72×10^{18}	6.18	76.17
10	384	180	4.67×10^{18}	7.75	82.92
11	384	210	5.24×10^{18}	8.70	86.44
12	384	240	5.67×10^{18}	9.42	92.25

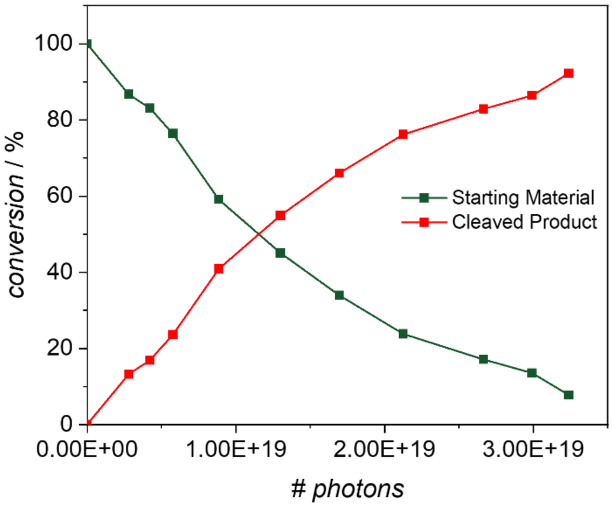

Figure S33: Reaction kinetics of compound **4**. To obtain approximately 25% consumption of the starting material, 1.93 µmol photons need to be deposited into the reaction system.

To obtain about 20-30% consumption of the starting material, 1.93 µmol photons (1.16×10^{18} photons) need to be deposited into the reaction system.

5.2.2 *Action Plot of Compound* **4**

The kinetic experiments in chapter 5.2.1 revealed that for each indicated wavelength, 1.93 µmol of photons need to be deposited in the reaction system. For action plot measurements, compound **4** was dissolved in MeCN with the total concentration being 80 µg mL^{-1} (150 µM). Sample solutions were freshly prepared every day and not kept overnight. The stock solution was prepared and bubbled with N_2 for 15 min, then 300 µL were withdrawn and added to capped laser vials (Supelco Vials, Merck) that had previously been flushed with N_2 for 5 min. For each indicated wavelength, three samples à 300 µL were irradiated, followed by filtration. 50 µL of the irradiation sample were mixed with 50 µL benzene (c = 1 mg mL^{-1} in MeCN) and analyzed via LC-MS to calculate the consumption of the starting material via comparison of the ration of the peak integrals of the starting material and the internal standard (**Table S6**).

Table S6: Detailed specifications of the laser and the calculations for the action plot of compound **4**.

Sample	*λ* / nm	*Pulse Energy* / µJ	*Irradiation time* / min	*Consumption of Starting Material* / %	*Standard Error* / %
1	310	600	02:35 s	18.9	
2	310	589	02:38 s	25.1	
3	310	623	02: 29 s	22.0	3.09
4	325	705	01:51 s	23.5	
5	325	749	01:45 s	23.7	
6	325	796	01:39 s	23.9	0.124
7	340	524	02:14 s	29.5	
8	340	530	02:13 s	32.6	
9	340	512	01:18 s	31.0	1.55
10	355	689	01:35 s	36.6	
11	355	712	01:32 s	37.0	
12	355	654	01:30 s	38.3	0.839
13	370	550	01:52 s	39.3	
14	370	573	01:48 s	36.5	
15	370	562	01:50 s	37.9	1.42
16	385	608	01:36 s	32.7	
17	385	592	01:39 s	31.3	
18	385	573	01:42 s	34.2	1.17
19	400	582	01:37 s	35.1	
20	400	555	01:41 s	34.7	
21	400	513	01:39 s	35.5	0.431
22	415	551	01:38 s	41.7	
23	415	520	01:40 s	42.1	
24	415	499	01:38 s	41.9	0.211
25	430	602	01:27 s	23.0	
26	430	610	01:25 s	22.7	
27	430	603	01:26 s	24.2	0.626
28	445	589	01:25 s	1.54	
29	445	570	01:28 s	4.23	
30	445	566	01:29 s	2.89	1.34
31	460	486	01:40 s	1.42	
32	460	490	01:39 s	2.42	
33	460	468	01:44 s	1.92	0.498
34	475	492	01:36 s	1.58	
35	475	497	01:35 s	1.91	
36	475	481	01:38 s	1.76	0.167
37	490	449	01:42 s	0.548	
38	490	467	01:38 s	3.15	
39	490	438	01:40 s	1.85	1.30
40	505	583	01:17 s	0	
41	505	567	01:18 s	0	

Sample	*λ / nm*	*Pulse Energy /* **µJ**	*Irradiation time /* **min**	*Consumption of Starting Material / %*	*Standard Error / %*
42	505	541	01:22 s	0	0
43	520	427	01:41 s	0	
44	520	430	01:40 s	0	
45	520	422	01:42 s	0	0

Once the wavelength-dependent reactivity was determined, a graph was plotted by overlaying the reactivity with the molar extinction spectrum, resulting in the chromophore's action plot (**Figure 2B** Main Article, **Figure S34**).

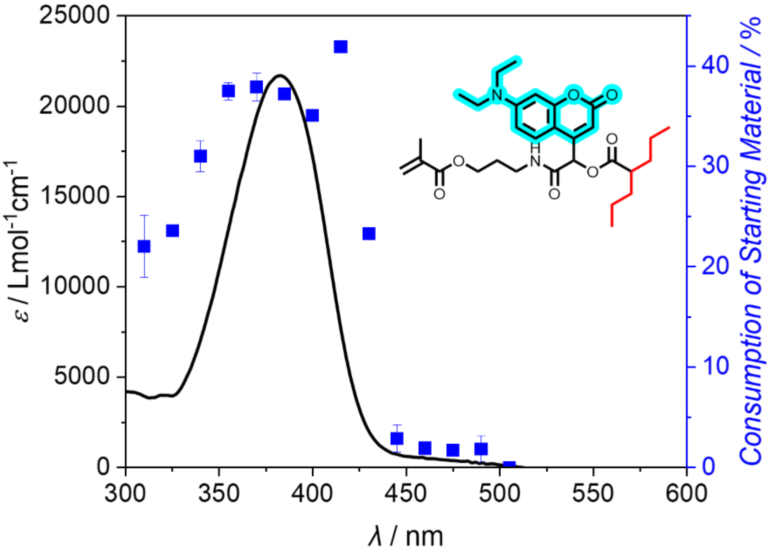

Figure S34: Molar extinction spectra of 3 overlayed with the action plot consumption of the starting material. For each indicated wavelength, 7.09 µmol photons were deposited into the reaction system (c_3 = 72 µM in MeCN).

6 LED Experiments

6.1 LED Emission Spectra and Irradiation Set Up

LED irradiation experiments were conducted using 72 µM DEA-Cou-PMA 2 (80 µg mL^{-1}) and 147 µM Per-PMA 3 (40 µg mL^{-1}) in a total volume of 5 mL MeCN in a crimp vial. The solution was filtered, equipped with a stir bar, sealed and degassed with Ar for 15 minutes. Both LEDs (405 nm and 505 nm) were purchased at Roithner LaserTechnik GmbH (5P4FCA 405 nm LED, B5B-433-B505 505 nm LED) and had their emission centred around 405 nm (**Figure S35A**, blue) and 505 nm (**Figure S35A**, green). Irradiation was carried out using a setup which was designed and manufactured by the University of Regensburg within the workshop of the Institute for Physical Chemistry at KIT (**Figure S35B**). The mixture was irradiated from the bottom of the vial under continuous stirring and constant temperature of 20 °C. Cooling was performed using a Lauda Alpha R8 thermostat.

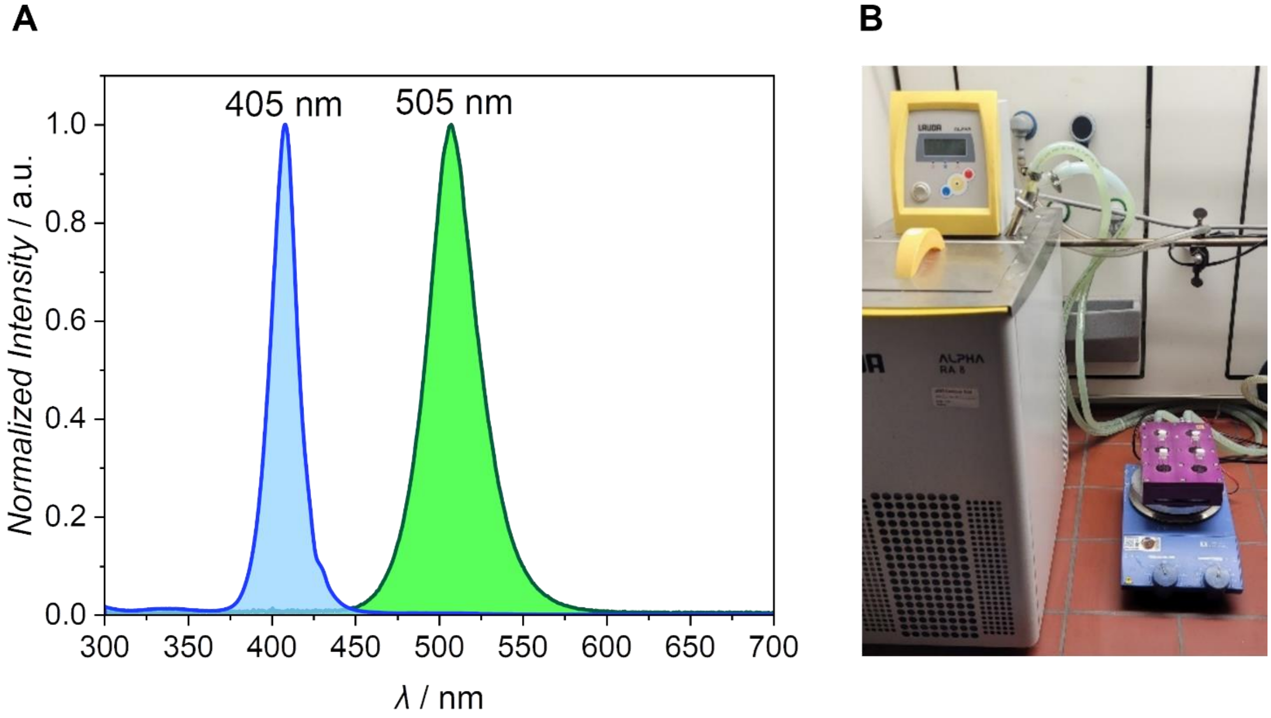

Figure S35: **A**: Normalized emission spectra of the two LEDs (405 nm, 0.2 mW and 505 nm, 40 mW) used for the sequential LED irradiation experiment. **B:** Irradiation setup with thermostat, magnetic stirrer, and crimp vial rack.

6.2 Results of the Sequential LED Experiment

At each indicated time slot, 3x 50 µL were taken out of the irradiated solution and analyzed via LC-MS. Prior to analysis, 50 µL benzene (c = 1 mg mL^{-1} in MeCN) was added as an internal standard to determine the consumption of both starting materials (**Table S7**).

Table S7: Results of the sequential LED experiment. The consumption of the starting materials was determined via LC-MS.

**λ / nm**	***Total irradiation time* / min**	***Consumption of 2* / %**	***Standard Error* / %**	***Consumption of 3* / %**	***Standard Error* / %**
405	20	16.6	1.21	0	-
405	40	22.9	0.758	0	-
405	60	30.4	1.06	0	-
405	80	44.5	0.0867	4.56	0.908
505	95	44.5	0.0867	14.4	0.635
505	120	44.5	0.0867	21.6	0.302
505	150	44.5	0.0867	25.3	0.942
505	180	44.5	0.0867	30.4	0.290
Dark	195	44.5	0.0867	30.4	0.290
Dark	210	44.5	0.0867	30.4	0.290
Dark	225	44.5	0.0867	30.4	0.290
505	235	44.5	0.0867	33.6	1.36
505	250	44.5	0.0867	39.9	1.127
405	265	62.6	0.757	42.2	0.837
405	280	73.3	0.603	46.6	1.36
405	305	83.1	0.690	47.9	1.35
405	320	89.0	0.1477	49.6	0.141
405	335	95.9	0.1014	53.1	1.88
505	360	95.9	0.1014	60.1	1.75
505	390	95.9	0.1014	78.8	1.33
505	420	95.9	0.1014	90.8	1.26

7 Proposed Mechanism of Photocleavage

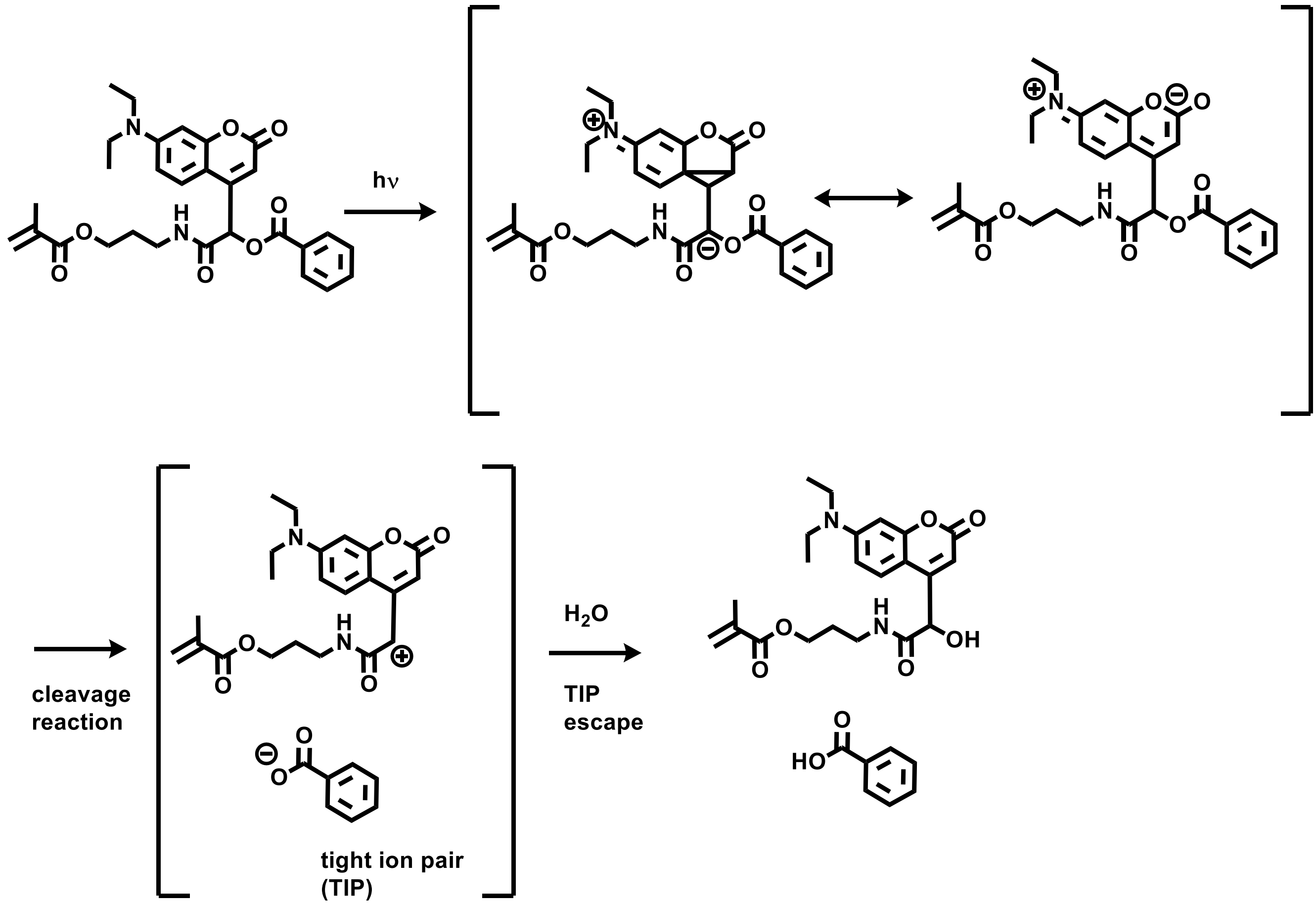

Scheme S6. The proposed mechanism[4] of the photocleavage reaction for (**2**), to yield the hydroxy-Passerini adduct, and the payload drug.

8 Calculation of Quantum Yield and Two-Photon Reactivity

Quantum Yield

The wavelength-resolved quantum yield plots were produced using data from the action plots and simple relations from the Beer-Lambert law as follows:

Knowing that $\Phi = \frac{Number\ of\ molecules\ converted}{Number\ of\ photons\ absorbed}$, where Φ is the quantum yield of conversion, we are able to derive the following equation:

$$\Phi = \frac{c \cdot V \cdot N_A \cdot \rho}{N_p \cdot (1 - 10^{-\varepsilon cl})}$$

Where c is concentration, V is volume, N_A is Avogadro's Number, ρ is conversion, N_p is total number of incident photons, ε is the molar extinction coefficient, and l is the path length of irradiation.

It is important to note that this derivation assumes that scatter is negligible, which at the concentrations described in the present study and with the size of molecule in the present study, is a valid approximation.

Two-Photon Reactivity

The rate of the two-photon absorption is described by the following equation:

$$R = \sigma_2 I^2$$

Where R is the rate of two-photon absorption, σ_2 is the two-photon absorption cross-section of the molecule, and I is the intensity of the incident light.

Given we irradiate a vial of diameter = 0.7 cm, we can calculate the intensity of photons. Taking the irradiation with the highest photon flux, (580 nm) with an irradiation time of 83 seconds with a 20 Hz laser with 5 ns pulses:

$$I = \frac{N_p}{t \cdot (\pi r^2)} = \frac{4.27 \times 10^{18}}{83 \cdot 5 \times 10^{-9} \cdot 20 \cdot (\pi \cdot 0.35^2)} = 1.31 \times 10^{24} \text{ photon}s \text{ cm}^{-2}\text{s}^{-1}$$

Using an experimentally determined value for σ_2 for perylene, 1.07 x 10^{-46} cm^4 s $photon^{-1}$ $molecule^{-1}$ from *B. Derkowska-Zielinska, Molecular Crystals and Liquid Crystals, 2018, 673, 149-154.*, we can calculate the rate of two photon absorption to be 184 photons per molecule per second. This accounts for only 3.6 x 10^{-13} % of the total number of photons; thus their contribution to the action plot is negligible.

9 NMR Study of Irradiation of 2

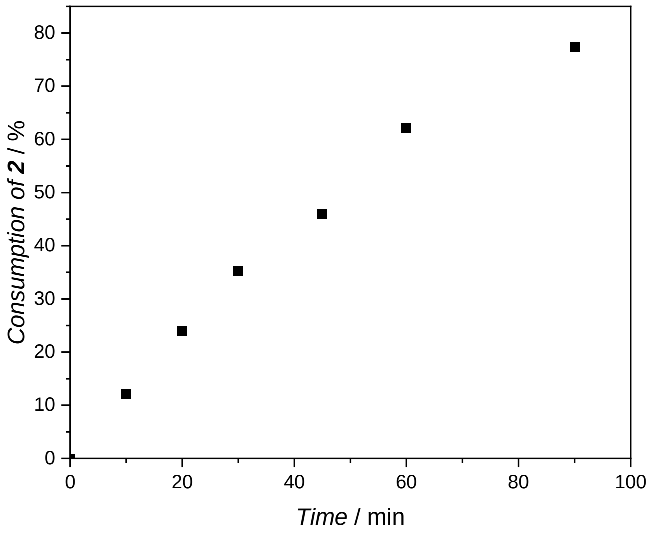

Figure S36: Consumption of **2** as a function of time, determined by measuring the integral of the resonance at δ = 8.07 ppm (**Figure S37**) relative to the methoxy protons of trimethoxy benzene.

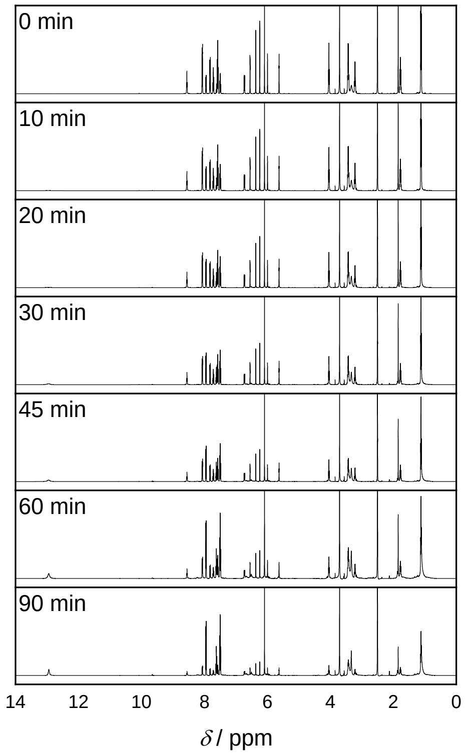

Figure S37: Stacked ^{1}H NMR spectra of **2** upon irradiation with 405 nm light, showing the successful cleavage of the benzoic acid moiety.

10 Control Irradiation Experiments

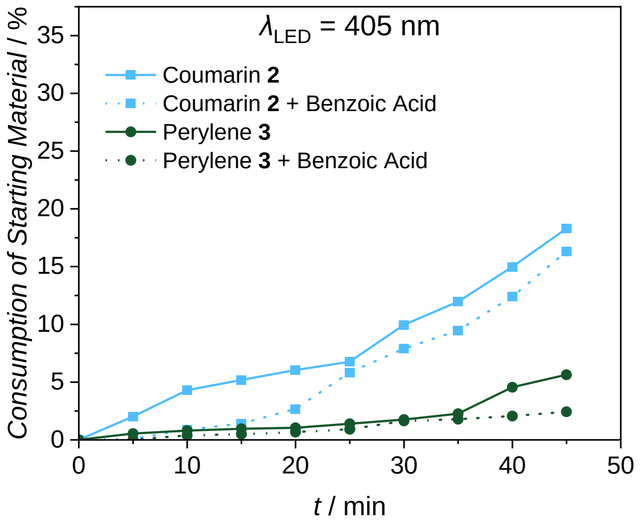

Figure S38: Consumption of the starting material **2** (72 µM) and **3** (147 µM) without (normal lines) and with benzoic acid (53 mM) (dashed lines) in an acetonitrile solution upon irradiation with 0.2 mW 405 nm light.

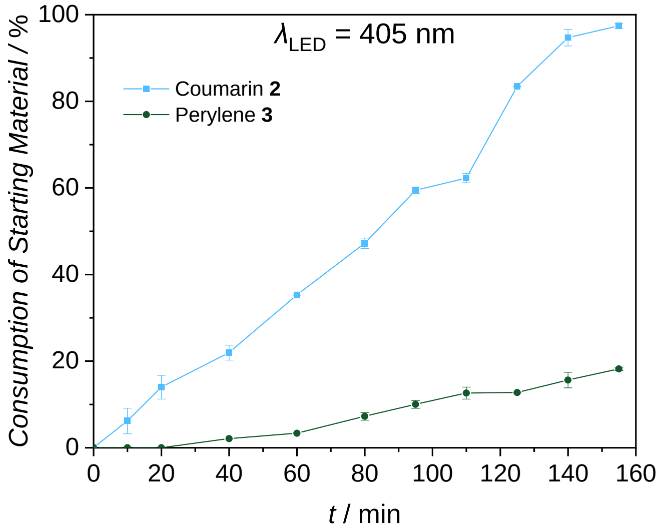

Figure S39: Consumption of the starting material **2** and **3** upon single wavelength irradiation (λ_{LED} = 405 nm, P = 0.2 mW).

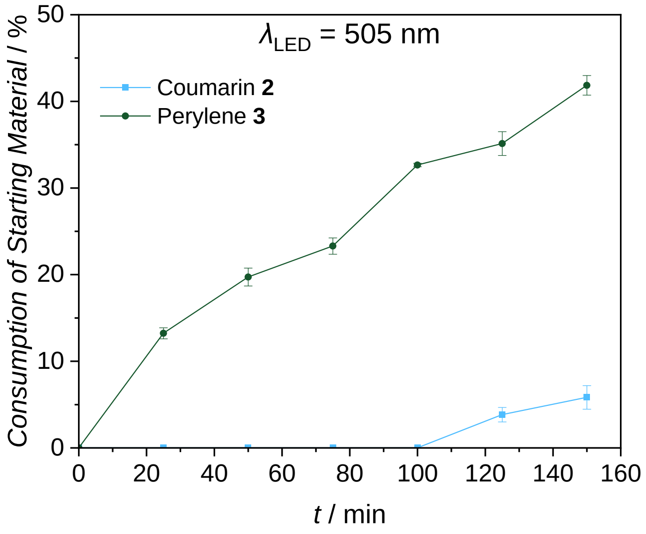

Figure S40: Consumption of the starting material **2** and **3** upon single wavelength irradiation (λ_{LED} = 505 nm, P = 40 mW).

11 References

[1] V. X. Truong, F. Li, J. S. Forsythe, *ACS Appl. Mater. Interfaces* **2017**, *9*, 32441-32445.

[2] refer to e. g. a) J. Bachmann, C. Petit, L. Michalek, Y. Catel, E. Blasco, J. P. Blinco, A.-N. Unterreiner, C. Barner-Kowollik, *ACS Macro Lett.* **2021**, *10*, 447-452; b) J. P. Menzel, B. B. Noble, J. P. Blinco, C. Barner-Kowollik, *Nat. Commun.* **2021**, *12*, 1691; c) B. T. Tuten, J. P. Menzel, K. Pahnke, J. P. Blinco, C. Barner-Kowollik, *Chem. Commun.* **2017**, *53*, 4501-4504; d) J. P. Menzel, B. B. Noble, A. Lauer, M. L. Coote, J. P. Blinco, C. Barner-Kowollik, *J. Am. Chem. Soc.* **2017**, *139*, 15812-15820.

[3] I. M. Irshadeen, S. L. Walden, M. Wegener, V. X. Truong, H. Frisch, J. P. Blinco, C. Barner-Kowollik, *J. Am. Chem. Soc.* **2021**, *143*, 21113-21126.

[4] a) R. Schmidt, D. Geissler, V. Hagen, J. Bendig, *J. Phys. Chem.* **2007**, *111*, 5768-5774; b) B. Schade, V. Hagen, R. Schmidt, R. Herbrich, E. Krause, T. Eckardt, J. Bendig, *J. Org. Chem.* **1999**, *64*, 9109-9117.
